# Exchange Current Densities of and Minority Carrier Fluxes to Catalyst Nanoparticles on Photoelectrodes from Intensity Modulated Photocurrent Spectroscopy

**DOI:** 10.1021/acsmeasuresciau.6c00287

**Published:** 2026-10-01

**Authors:** Max Nusshör, Eric Hinssen, Maximilian Diecke, Erica A. Schmitt, Matthias M. May

**Affiliations:** † Institute of Physical and Theoretical Chemistry, Universität Tübingen, Tübingen 72076, Germany; ‡ Center for Light-Matter Interaction, Sensors and Analytics LISA+, Universität Tübingen, Tübingen 72076, Germany

**Keywords:** exchange current density, electrochemistry, intensity modulated photocurrent spectroscopy, nanoparticles, photoelectrocatalysis

## Abstract

The photoelectrochemical production of chemicals is a promising approach to replace greenhouse gas-emitting production processes. Here, an illuminated semiconducting photoelectrode provides the electrochemical potential difference to drive the targeted electrochemical reaction. Since the catalytic activity of the pristine surface is typically limited, co-catalysts are integrated on the electrochemical interface to reduce overpotentials and their form of nanoparticles avoids parasitic light absorption. The classical approach for the choice of the catalyst is based on the activity of the bulk metal electrode, but a quantification of the catalytic activity of the deposited nanoparticle catalysts is usually not done. This can be attributed to the fact that, for photoelectrodes, standard electrochemical methods do not allow for a direct evaluation of the catalytic activity of the co-catalysts. Using intensity modulated photocurrent spectroscopy, we develop a method to quantify the catalytic activity of nanoparticle catalysts on photoelectrodes. We obtain Tafel plots, analogously to the quantification of the catalytic activity of bulk metal electrodes. From that, we derive the exchange current density of the photoelectrode. Therefore, a comparison of the catalytic activity with the bulk material of the nanoparticles and the evaluation of catalytic activities of different nanoparticle materials become possible. Furthermore, we obtain plots correlating the light-generated minority carrier flux with the band bending in the semiconducting material. These plots give direct insight into the ability of the semiconducting material to generate and separate electron–hole pairs and helps to identify performance bottlenecks of photoelectrochemical interfaces.

## Graphical abstract

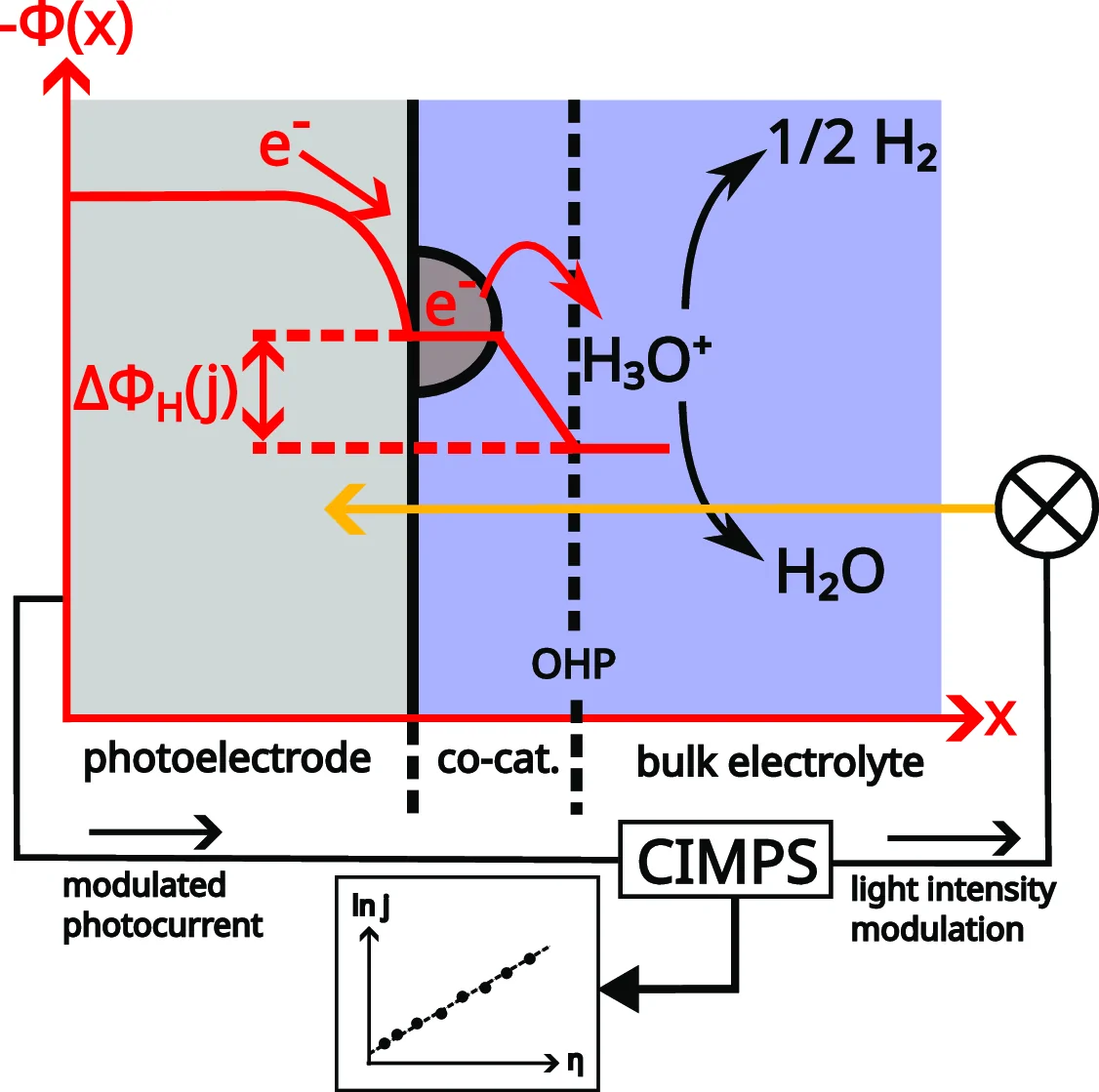

## 1 Introduction

The application of semiconducting photoelectrodes for energy or matter conversion and storage using solar irradiation is a scientifically intriguing and potentially economical approach.^1^ One of the important characteristics for efficient photoelectrodes, irrespective of the target product, is the overpotential associated with the desired photoelectrochemical reaction at the interface between photoelectrode and electrolyte. As most photoabsorber materials are comparatively poor electrocatalysts, efficient devices for photoelectrocatalysis require the introduction of co-catalysts on the surface of the semiconducting material.^2,3^ These co-catalysts typically have to be integrated into the device in the form of weakly interconnected nanoparticles to avoid both, parasitic light absorption and unfavorable band alignment between photoabsorber and catalyst.^4–6^

In the case of the hydrogen evolution reaction (HER), one often uses nanoparticles of metals of the platinum group, since these metals are in their bulk form known to be catalytically very active for the HER.^7^ This high catalytic activity is then typically also found for nanoparticles deposited on the surface of photoelectrodes.^6^ While the transfer of catalytic properties from bulk material to nanoparticles works qualitatively in the case of platinum group nanoparticles, there has not yet been a direct method to quantify the catalytic properties of the functionalized surfaces available. Here, the complexity of the photoelectrode system does not allow one to separate, by standard electrochemical measurements such as Tafel plots, the kinetic overpotential associated with the photoelectrochemical reaction from overpotentials associated with charge separation and transfer processes in the semiconducting material.^8,9^

Kühne and Schefold proposed to use light-induced flatband shifts determined with the help of Mott-Schottky plots to estimate the catalytic parameters of layers of different metals covering *p*-InP substrates for the HER.^10^ While they were able to estimate exchange current densities for the HER for different metals, their obtained values did not depend on the amount of deposited metal co-catalyst. This finding is quite odd since for the amounts deposited in their work, one would expect nanoparticular metal island formations.^11^ Increasing the amount of deposited metal, these islands should cover more and more of the surface of the semiconducting substrate and therefore increase the determined exchange current density. Furthermore, their method seems to struggle with highly efficient co-catalyst materials. For platinum, they presented only very few data points leading to questionable accuracy in the fit used to determine the exchange current density. Later, the same authors themselves questioned the accuracy of their method^12^ due to potential common errors in the Mott–Schottky measurements.^13^ In the same paper, they proposed to use electrochemical impedance spectroscopy (EIS) to obtain the exchange current densities.^12^ In this work they now obtained, as expected, exchange current densities that varied with the amount of co-catalyst deposited. Compared to literature reporting efficient *p*-InP photocathodes covered with metal co-catalyst of similar loading,^11^ their values for the exchange current densities seem to be too low, though. Since they fitted the EIS data to a model to obtain the exchange current density, there is the possibility for a misinterpretation of the data due to an ambiguous model selection. Especially for illuminated semiconducting electrodes, EIS models may become quite complex and qualitatively (and then of course also quantitative) wrong conclusions may follow easily.^9^

In this work, we show that intensity modulated photocurrent spectroscopy (IMPS) allows one to get an estimate for the exchange current density of a photoelectrode covered with metal nanoparticles without relying on a specific model for fitting the data. Thus, a quantitative analysis of the catalytic properties of photoelectrode surfaces functionalized with co-catalysts becomes possible. Furthermore, using the obtained catalytic parameters of the co-catalyst, the light-generated minority carrier flux can be correlated to the band bending in the semiconducting material. This allows one to gain direct insight in the ability of the semiconducting material to generate and separate electron–hole pairs. Furthermore, our results also demonstrate limitations in the applicability of the Gärtner flux model.

## 2 Method Development

### 2.1 Intensity Modulated Photocurrent Spectroscopy

IMPS is an impedance-based method for investigating photovoltaic devices such as photoelectrodes. In contrast to conventional EIS, where the applied bias potential is perturbed sinusoidally and the resulting alternating current is measured (or vice versa), in IMPS the potential is held constant, but the illumination intensity, Γ, is varied sinusoidally around a fixed DC value, Γ_DC_

1 $$\Gamma \left(t\right)=\Gamma_{DC}+\mathrm{\Gamma ̃}\sin\left(\omega t\right)$$

where ω is the angular frequency of the perturbation and Γ̃ is the AC amplitude of the perturbation. As in impedance spectroscopy, also for IMPS the resulting alternating current, j̃, is measured. To aid the understanding of the following discussion, a simplistic sketch describing the processes relevant to understand the IMPS response is shown in Figure 1.

**Figure 1:**
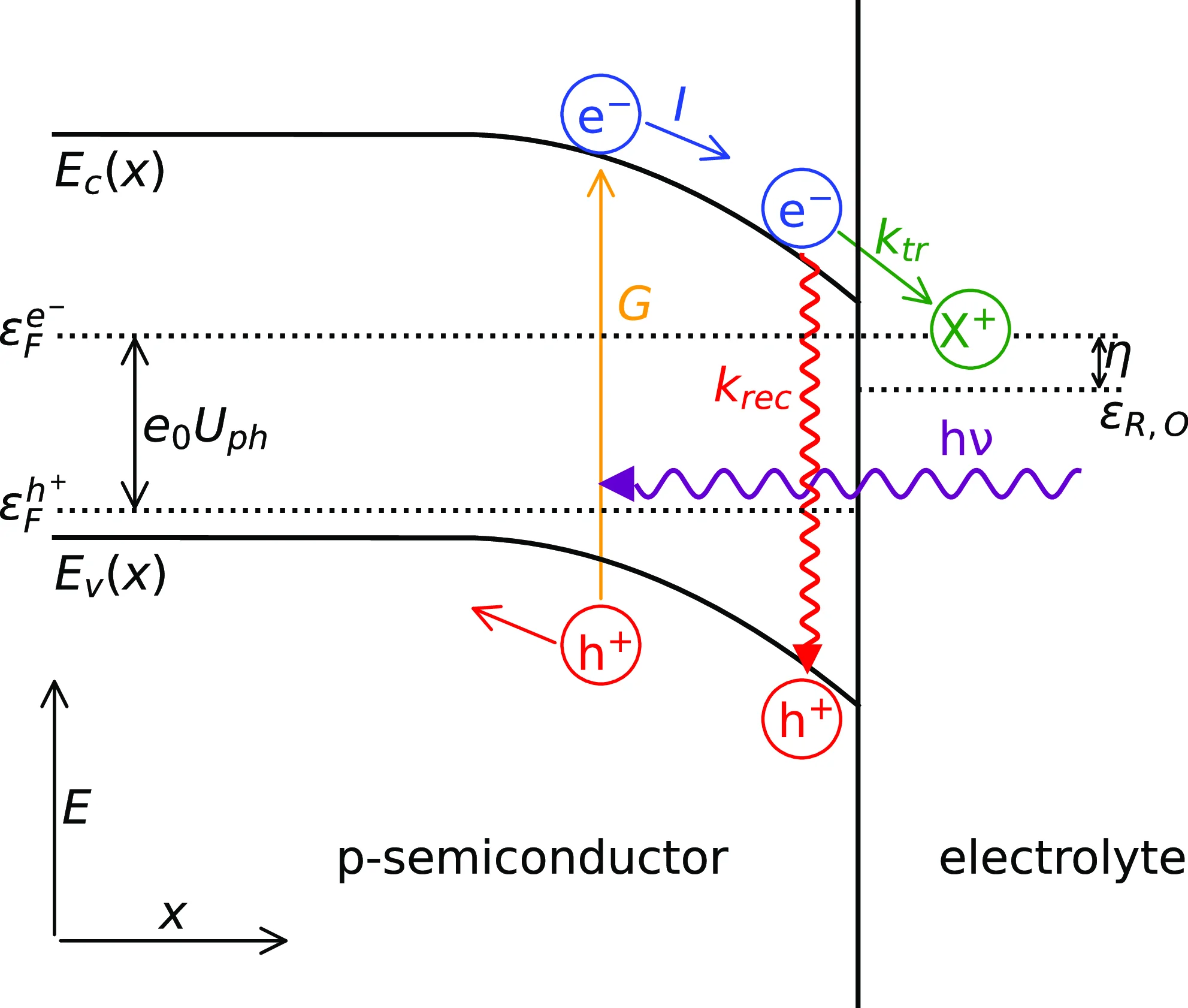
Simplistic sketch of the processes relevant to understand the IMPS response of a p-type semiconductor photocathode. The valence band edge, *E*_*v*_(*x*), and the conduction band edge, *E*_*c*_(*x*), are shown. Band bending is indicated by the dependence of the band edges on position *x*. The p-type semiconductor is assumed to be in depletion layer mode as typical for photoelectrodes without dedicated charge-selective contact.^14^ Photons impinging the surface of the semiconducting material are implied by the purple arrow. Absorption of the photons in the semiconducting material leads to electron–hole pair generation, *G*. Separation of these electron–hole pairs leads to a minority carrier (electrons) flux, *I*, directed to the surface of the semiconducting material. At the surface, these electrons may either recombine with majority carrier (holes) present at the surface (*k*_rec_) or be transferred to an oxidised species in the electrolyte, driving a photoelectrochemical reduction reaction (*k*_tr_). Also indicated are the (quasi-) Fermi levels of the electrons, $\varepsilon_{F}^{e^{-}}$ , and the holes, $\varepsilon_{F}^{h^{+}}$ , as well as the redox level in the electrolyte ε_R,O_. The photovoltage *U*_ph_ corresponds to the difference in the quasi-Fermi levels of electrons and holes: $e_{0}U_{ph}=\varepsilon_{F}^{e^{-}}-\varepsilon_{F}^{h^{+}}$ . The overpotential η is defined as the difference of electron quasi-Fermi level and redox level: $e_{0}\eta =\varepsilon_{F}^{e^{-}}-\varepsilon_{R,O}$ . For further clarification, see also the discussion in the text. For a photoanode, an analogous sketch may be drawn reversing the direction of band bending and the roles of electrons and holes. Inspired by ref 15.

IMPS has been widely used to determine charge transfer constants of photoelectrochemical reactions and surface electron–hole recombination rates of bare or with co-catalyst decorated photoelectrode surfaces.^16–22^ While this approach is relatively straightforward for photoelectrodes with a bare surface, two issues arise when considering photoelectrodes decorated with a co-catalyst. Firstly, for an efficient photoelectrochemical system, standard IMPS evaluation of the charge transfer constant is not possible, anymore, since the rate constant is determined via the so-called recombination semicircle.^15,23^ This semicircle is only observable in the IMPS spectrum if the electron–hole pair recombination at the surface of the photoelectrode proceeds in a considerable rate compared to the charge transfer rate. In Figure 2, an exemplary IMPS spectrum is shown for clarification. The charge transfer rate, *v*_tr_, and the recombination rate, *v*_rec_, are modelled using pseudo first order rate equations^15^

2 $$v_{tr}=k_{tr}n_{s}$$

and

3 $$v_{rec}=k_{rec}n_{s}$$

**Figure 2:**
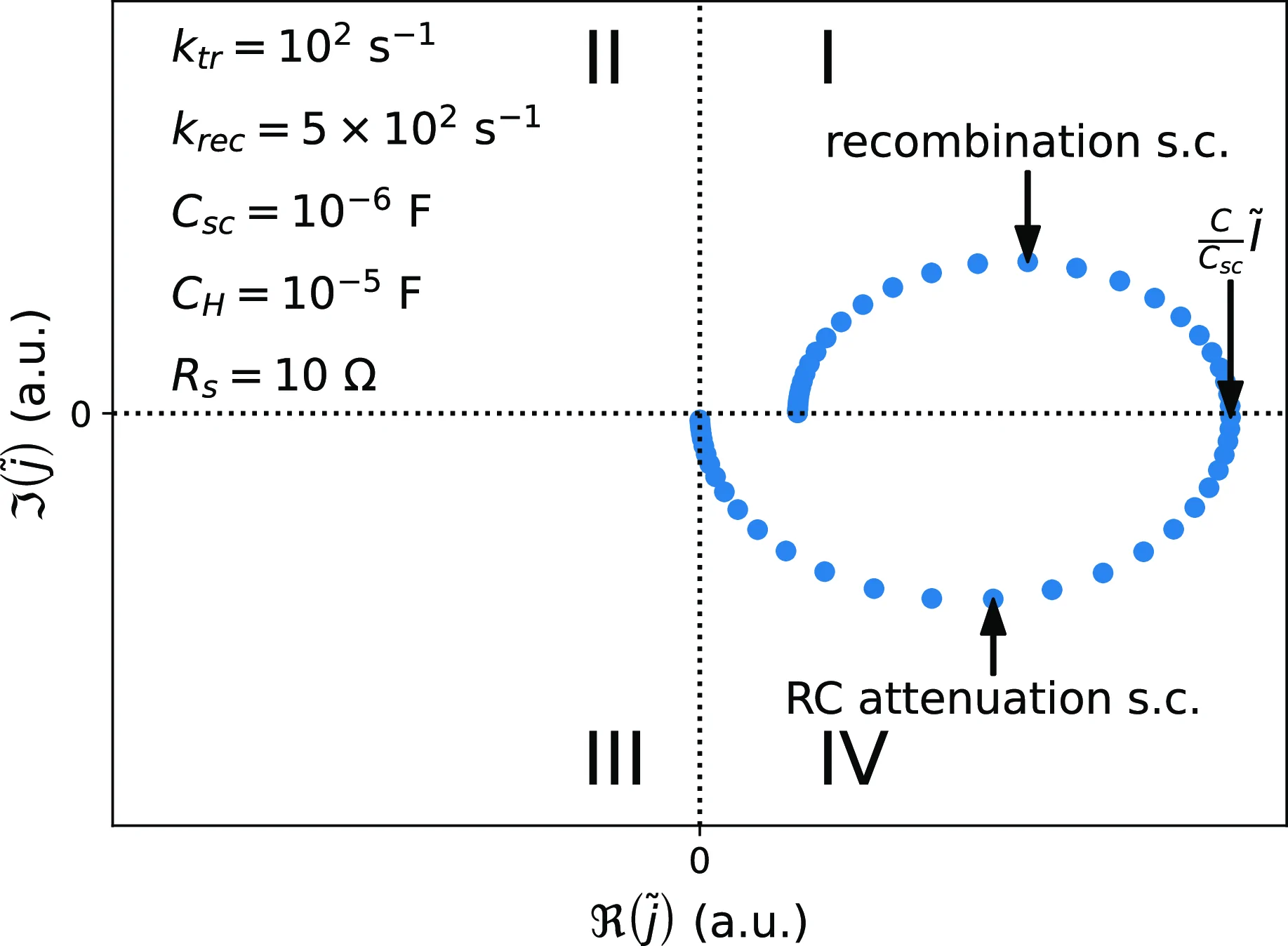
Exemplary, based on concepts described in ref 15, simulated IMPS spectrum plotting the imaginary part of the AC component of the photocurrent response, I(j̃) , against the corresponding real part, R(j̃) . Parameter values used for the simulation are given in the top left corner. These parameters are the charge transfer constant, *k*_tr_, the recombination rate constant, *k*_rec_, the capacitance of the semiconducting material, *C*_sc_, the capacitance of the Helmholtz layer, *C*_*H*_, and the series resistance of the electrochemical cell, *R*_*s*_. The quadrants of the complex plane are highlighted with roman numerals. The so-called recombination semicircle (s.c.) is located in the first quadrant, while the so-called RC attenuation semicircle is located in the fourth quadrant. Also highlighted is the right intercept with the real axis of the IMPS response giving the AC minority carrier flux, Ĩ, scaled by $\frac{C}{C_{sc}}$ , where *C* is the total capacitance of the photoelectrode (compare eq 9).

Here, *n*_*s*_ is the amount of minority carriers at the surface of the photoelectrode and *k*_tr_ and *k*_rec_ are the pseudo first order rate constants describing charge transfer and electron–hole pair annihilation, respectively. Note that we are considering a photocathode in the following, so the minority carriers are electrons. The following reasoning is of course directly transferable to the case of a photoanode making appropriate sign changes.

In the following, we discuss occurrence and shapes of semicircles arising in IMPS spectra, linking them to quantities of interest like the charge transfer rate and the interfacial capacitance. The occurrence of the recombination semicircle can be linked to the approximate condition *k*_rec_ ≳ 0.1 × *k*_tr_. This may, however, not be expected to still hold for a photoelectrode covered with an efficient co-catalyst. Then, the determination of the rate constants by the standard IMPS procedure is not possible, anymore. It has been noted in the literature though, that in the case *k*_tr_ ≫ *k*_rec_, a second semicircle in the fourth quadrant of the complex plane may arise with a time constant equal to *k*_tr_^–1^ if the capacitance of the semiconductor, *C*_sc_, is comparable in size to the Helmholtz capacitance, *C*_*H*_.^15,24^ In this case, the determination of *k*_tr_ may still be possible, although *k*_rec_ ≤ 0.1 × *k*_tr_ holds. For this, $k_{tr}\ll {\left(R_{s}C\right)}^{-1}$ must still hold to separate the additional semicircle from the RC attenuation semicircle.^15^ Here, *R*_*s*_ is the series resistance of the electrochemical cell and $C={\left(\frac{1}{C_{sc}}+\frac{1}{C_{H}}\right)}^{-1}$ is the total capacitance of the photoelectrode. Especially for semiconductor–metal contacts, this will likely not be fulfilled since there, *k*_tr_ is expected to be many orders of magnitude larger compared to the values of *k*_tr_ = 1 s^–1^–10^3^ s^–1^ ^17–22,25^ expected for a semiconductor electrolyte contact.^26^ ${\left(R_{s}C\right)}^{-1}$ may be expected to be in the order of 10^4^ s^–1^–10^6^ s^–1^.^15,17,21,25^

Furthermore, when a co-catalyst decorates the surface of a photoelectrode, it is not obvious at all, if one then measures the rate constant for the charge transfer from semiconducting material to co-catalyst, *k*_tr_^sc,cc^, or from co-catalyst to electrolyte species, *k*_tr_^cc,el^, or a weighted mixture of both. This issue has only been discussed tentatively in literature, but was not further analysed.^21^ To highlight this issue further, we consider the basic equations proposed by Peter and coworkers^15^ from which the IMPS response for a photocathode follow, extended by a term describing the charge *Q*_cc_ stored in the co-catalyst layer. Furthermore, we assume no potential drop in the co-catalyst layer (either metallic co-catalyst or nano-particular with dimensions smaller than the Debye length of the co-catalyst material). Denoting the Fourier component of a time-dependent quantity *x*(*t*) with x̃, we obtain

4 $$i\omega {ñ}_{s}=Ĩ-k_{tr}^{sc,cc}{ñ}_{s}-k_{rec}{ñ}_{s}$$

5 $$i\omega {\mathrm{Q̃}}_{sc}=Ĩ-k_{rec}{ñ}_{s}+\mathrm{j̃}$$

6 $$i\omega {\mathrm{Q̃}}_{cc}=-k_{tr}^{sc,cc}{ñ}_{s}-k_{tr}^{cc,el}{\mathrm{Q̃}}_{cc}$$

and

7 $$i\omega {\mathrm{Q̃}}_{H}=k_{tr}^{cc,el}{\mathrm{Q̃}}_{cc}-\mathrm{j̃}$$

here, *I* is the minority carrier flux to the surface of the photoelectrode, *Q*_sc_ the charge stored in the depletion layer, *j* the current flowing through the external circuit (measured by the potentiostat), and *Q*_*H*_ the charge stored in the Helmholtz layer. Note that in this treatment, possible AC components of rate constants are neglected.^9,15^ Furthermore, note that *n*_*s*_ describes now the amount of surface electrons multiplied by the elementary charge, *e*_0_. To solve the system of equations, one imposes the potentiostatic condition of the IMPS measurement protocol^15^

8 $$\frac{{\mathrm{Q̃}}_{sc}}{C_{sc}}-\frac{{\mathrm{Q̃}}_{H}}{C_{H}}+\mathrm{j̃}R_{s}=0$$

Note that the rate of the charge transfer from the co-catalyst to the electrolyte species, *k*_tr_^cc,el^, comes into play in the second part of the potentiostatic condition only (through ${\mathrm{Q̃}}_{H}$ ). This part is damped by the Helmholtz capacitance, which is typically at least an order of magnitude larger than the depletion layer capacitance of the semiconductor material.^27,28^ Thus, the second part of the potentiostatic condition may be expected to contribute only a minor part to the response. The charge transfer from the co-catalyst to the electrolyte may therefore not be significantly probed by the IMPS method.

To circumvent these issues, we propose a different approach for using IMPS to evaluate the efficiency of a co-catalyst decorating a photoelectrode surface. Our method is based on the observation that the magnitude of the IMPS response should show a one-to-one correspondence with band bending in the photoelectrode. When plotting the imaginary part of j̃ against the real part of j̃, the magnitude of the IMPS response is defined here as the right intercept (r.i.) of the response with the real axis. Equivalently, this corresponds to the maximum real part of j̃ (see Figure 2). Here, charge transfer and recombination are frozen out, while RC attenuation is still negligible. Following the treatment by Peter and coworkers, j̃ at this point is given by^15^

9 $$\mathrm{j̃}\left(r.i.\right)=\frac{C}{C_{sc}}Ĩ$$

The AC component of the minority carrier flux, Ĩ, may be modelled in the simplest case by the Gärtner flux^29^

10 $$Ĩ=e_{0}\mathrm{\Phi ̃}\left[1-\frac{\exp\left(-\alpha W_{dl}\right)}{1+\alpha L_{n}}\right]$$

here, Φ̃ is the AC component of the impinging photon flux (monochromatic illumination assumed), α is the absorption coefficient of the semiconducting material, *L*_*n*_ is the minority carrier diffusion length in the bulk of the semiconductor, and *W*_dl_ is the depletion layer width. Φ̃ is directly related to the AC component of the illumination intensity by $\mathrm{\Phi ̃}=\frac{\mathrm{\Gamma ̃}}{h\nu }$ , where *h* is Planck’s constant and ν the light frequency of the monochromatic illumination. Note that a more careful consideration has to take into account a possible frequency dependence of the Gärtner flux due to the minority carrier lifetime, τ_*n*_.^30^ Considering the continuity equation of the minority carriers in the bulk in the Fourier domain

11 iωñ(x)=Dnd2ñdx2(x)+G̃(x)−ñ(x)τn⇒ñ(x)(iω+τn−1)=Dnd2ñdx2(x)+G̃(x)

with the diffusion coefficient, *D*_*n*_, and the light-driven electron–hole pair generation rate, G̃, it is clear that this frequency dependence will not play a role for ω≪τ_*n*_^–1^. G̃ is not expected to be dependent on the frequency of the AC illumination intensity since the generation of electron–hole pairs by light absorption and their thermalisation to the band edges proceeds in a time span of picoseconds^31^ and is thus a far too fast a process to be resolved by IMPS. Considering, for example, typical values of III–V semiconducting materials for τ_*n*_ in the order of ns to μs,^32^ this translates to the condition $\omega ≳10^{6}\frac{rad}{s}$ for a relevant contribution to the AC minority carrier flux. For silicon as an example on the other hand, lifetimes may range up to 1 ms^33^ and there, the frequency dependence due to the carrier lifetime may play a role for angular frequencies no larger than $10^{3}\frac{rad}{s}$ .

Interestingly, this may be an issue when estimating charge transfer and recombination rate constants of bare semiconducting surfaces using the standard IMPS procedure,^15,23^ since these rate constants can range up to 1000 s^–1^ ^21,25^ and may therefore be probed in a similar frequency range. In this case, the estimation of the rate constants may be perturbed by the frequency dependence of the minority carrier flux. For the case we are considering here, where the photoelectrode system is an efficient system due to a co-catalyst (*k*_tr_ ≫ *k*_rec_), this issue will not arise, since as stated above, the rate constants are not probed then, anyway, and the magnitude of the IMPS response as defined above can be determined by taking the value of the IMPS response for ω → 0. Since we are considering InP, a III–V semiconductor with minority carrier lifetimes in the ns region,^34^ as exemplary photoelectrode material in this work, we may in any case regard the DC Gärtner flux as reasonable approximation for the AC minority carrier flux by simply replacing the DC photon flux, Φ, with the AC photon flux, Φ̃. Note also that for non-monochromatic illumination, the total AC minority carrier flux will simply correspond to the integral of the monochromatic AC minority carrier flux over the whole spectral region of the non-monochromatic illumination source.

Importantly, the Gärtner flux is directly related to the band bending, Δϕ_sc_, due to the width of the depletion layer, *W*_dl_. In the depletion layer approximation, one finds^27,28^

12 $$W_{dl}=\sqrt{\frac{2\varepsilon_{sc}}{e_{0}N}\left(\Delta \phi_{sc}-\frac{k_{B}T}{e_{0}}\right)}$$

with the dielectric constant, ε_sc_, of the semiconducting material, the doping concentration, *N*, the Boltzmann constant, *k*_*B*_, and the absolute temperature, *T*. Thus, the depletion layer width increases with increasing band bending and this in turn leads to a monotonic increase of the AC Gärtner flux (eq 10) with band bending. So, we find that the AC minority carrier flux, Ĩ, shows a one-to-one correspondence with the DC band bending, Δϕ_sc_. Note that with IMPS, we can only find the AC minority carrier flux scaled by the total geometric capacitance, divided by the depletion layer capacitance (see eq 9)­

13 $$\frac{C}{C_{sc}}Ĩ=\frac{C_{H}}{C_{sc}+C_{H}}Ĩ$$

In general, the scaling factor will be close to one since typically *C*_sc_ ≪ *C*_*H*_ unless the semiconducting material is highly doped.^27,28^ Even in the case of a highly doped semiconductor, the scaling factor is expected to increase with increasing band bending, since the depletion layer capacitance decreases with increasing band bending.^28^ Also, note that a homogeneously doped photoelectrode is typically operated in depletion mode. Even for a highly doped semiconducting material, the capacitance in the depletion mode will very likely still be smaller than the Helmholtz capacitance. To stress this point, we calculate the capacitance in the depletion layer approximation for a *p*-InP electrode doped with a doping concentration of *N*_*A*_ = 4 × 10^18^ cm^–3^ as used in our study. One obtains with the thermal voltage *V*_th_ ≈ 25 mV, the relative permittivity of InP ε_sc_ = 12.5 ^35^ and the vacuum permittivity ε_0_ ^27^

14 $$C_{sc}=\sqrt{\frac{\varepsilon_{0}\varepsilon_{r}N_{A}e_{0}}{2}}{\left(\Delta \phi_{sc}-V_{th}\right)}^{-1/2}≃\frac{0.6\frac{\mu F}{cm^{2}}}{{\left(\Delta \phi_{sc}-V_{th}\right)}^{1/2}}$$

Inserting explicit values for the potential drop in the semiconducting material, one obtains $C_{sc}≃3.8\frac{\mu F}{cm^{2}}$ for Δϕ_sc_ = 50 mV and $C_{sc}≃2.2\frac{\mu F}{cm^{2}}$ for Δϕ_sc_ = 100 mV. Comparing with typical values for the Helmholtz capacitance of $C_{H}=10-80\frac{\mu F}{cm^{2}}$ ,^27^ the capacitance of the *p*-InP samples in depletion mode may only be comparable to the Helmholtz capacitance very close to the flatband potential and will otherwise be at least an order of magnitude smaller than the Helmholtz capacitance. Thus, we conclude that the influence of the Helmholtz capacitance can be neglected for our samples anyway. Note that in principle the Helmholtz capacitance may vary with applied bias potential^27,36^ and this could in principle affect the scaling factor (if the semiconductor capacitance would be comparable to the Helmholtz capacitance for a considerable range of applied bias potentials).

Thus, the scaling factor and the AC minority carrier flux should increase with band bending meaning that the total r.i. of the IMPS response (eq 9) should increase with band bending. While the Gärtner flux has already been used to fit^37^ and interpret^38^ IMPS data, our approach does not require the assumption of a specific relationship between the minority carrier flux and band bending to hold. We only propose that the minority carrier flux as determined by IMPS (scaled by the discussed prefactor) shows a one-to-one correspondence with band bending in the photoelectrode. This will be true more generally than assuming the specific form of the Gärtner flux, which relies on the assumption of perfect electron–hole pair separation in the depletion layer.^29^

It is of interest to discuss possible effects leading to a failure of the assumption that the minority carrier flux depends only on band bending. One could envisage nonlinearities in the bulk recombination for large illumination intensities (e.g. concentrated sunlight) due to high injection conditions. Assuming low injection conditions (moderate illumination intensities), one could envisage possible nonlinearities of the recombination in the depletion layer due to e.g. the Shockley-Read-Hall (SRH) recombination process.^39^ Then, the minority carrier flux may in principle be dependent on the DC illumination intensity, since the DC carrier densities are determined by the DC illumination intensity. For this to be relevant, the nonlinearities in the SRH recombination would have to be large compared to the generated AC minority carrier flux. One may argue that recombination in the depletion region will be of importance especially close to flatband conditions. For large band bending, the electric field in the depletion region is large and thus separation of electron–hole pairs becomes faster,^14^ leading to a reduced importance of the recombination in the depletion layer in determining the minority carrier flux. Note that close to the flatband potential, the majority carrier concentration is of similar magnitude in the depletion layer compared to the bulk. Then, as in the bulk, the SRH recombination becomes independent of the majority carrier concentration and a recombination term linear in the minority carrier concentration follows. One can thus conclude, that relevant deviations from the assumption of a bijective relationship between minority carrier flux and band bending are, for low injection conditions, only likely for intermediate band bending. If a deviation occurred, then it would depend on the relative importance of SRH recombination when compared to the separation efficiency of electron–hole pairs in this region. Since SRH recombination rates as well as drift and diffusion lengths are dependent on both the type and the quality of the material, the occurrence of nonlinearities of the type discussed here is heavily dependent on the composition and fabrication of the photoelectrode under investigation.

Note that we have neglected a possible influence of surface states up to now. As shown by Ponomarev and Peter, the presence of surface states can alter the quantitative and also qualitative behaviour of the IMPS spectra if the capacitance of the surface states is comparable to the Helmholtz capacitance.^15^ If the time constants corresponding to filling of the surface states and the recombination semicircle are of comparable size, two convoluted semicircles arise and the spectra become more complicated to analyse. Then, the total r.i. of the IMPS response may contain a contribution due to the capacitance of the surface states and the conclusion, based on our assumptions, that the right intercept increases with band bending does not necessarily have to hold any more.

### 2.2 Tafel Plots

One goal of our method is to determine the catalytic efficiency of co-catalysts decorating the surface of photoelectrodes. Although this will in general not be true for photoelectrodes,^40^ we assume a Butler-Volmer-type behaviour^41^ to hold for the dependence of the electric current density, *j*, on the overpotential, η, dropping in the Helmholtz layer

15 $$j=j_{0}\left[\exp\left(\beta f\eta \right)-\exp\left(-\alpha f\eta \right)\right]$$

here, *j*_0_ is the exchange current density, α and β are the cathodic and anodic charge transfer coefficient, where for a one-step electrochemical reaction 0 ≤ α, β ≤ 1, α + β = 1 holds. Furthermore, $f=\frac{F}{RT}$ , where *F* is the Faraday constant, *R* is the ideal gas constant and *T* is the absolute temperature.

Note that the assumption of a Butler-Volmer-type behaviour for a photoelectrode decorated with a co-catalyst can be justified if one assumes that the co-catalyst is of metallic nature and that most of the faradaic current flows through the co-catalyst. In the present case, this assumption is justified by the high minority carrier diffusion lengths on the order of several μm in InP^42,43^ when compared to the spacing between the co-catalyst nanoparticles. This can be understood immediately when noticing that the change in electron density in a metal with varying applied conditions is negligible. Thus, a change in electrochemical kinetics in the case of a metal catalyst can only be induced by a change in the rate constant. Assuming, as usual in electrochemistry, the rate constant to be exponentially dependent on the potential drop in the Helmholtz layer, a Butler-Volmer-type behaviour for an electrochemical current flowing through a metal catalyst follows directly. This holds regardless if the metal is present in bulk form or as nanoparticles.

For considerable cathodic polarisation of the interface (η ≤ −0.1 V), the anodic part may be neglected and

16 $$j≃-j_{0}\;\exp\left(-\alpha f\eta \right)$$

holds. For a photocathode, for which these relations hold, *j*_0_ and α would directly describe the catalytic properties of the interface for a photoelectrochemical reduction at finite rate. To obtain these parameters for metal electrodes, the method of Tafel plots is well-established. Here, one plots the logarithm of the absolute value of the current density against the applied overpotential. For considerable cathodic overpotential, such that eq 16 holds, a linear relation is found

17 $$\ln\left(|j|\right)=\ln\left(j_{0}\right)-\alpha f\eta$$

The (logarithm of the) exchange current density may then be obtained by the linear extrapolation η → 0^–^, while α is obtained by the slope of the linear relation. The ansatz described above works well for metal electrodes, yet for semiconducting electrodes, the issue arises that η is not readily obtainable. For a metal electrode, the applied potential, *U*_bias_, measured against a reference potential, *U*_ref_, is dropped in the Helmholtz layer, (Δϕ_*H*_(*j*)), and over the series resistance, *R*_*s*_, of the electrochemical cell

18 $$U_{bias}\left(j\right)-U_{ref}=\Delta \phi_{H}\left(j\right)+jR_{s}$$

The overpotential is a function of the current density

19 $$\eta \left(j\right)=\Delta \phi_{H}\left(j\right)-\Delta \phi_{H}^{eq}$$

here, Δϕ_*H*_^eq^ is the potential drop in the Helmholtz layer at equilibrium conditions, where *U*_bias_ = *U*_bias_^eq^ and *j* = 0 holds. If one determines the series resistance via electrochemical impedance spectroscopy for example, the overpotential corresponding to a given current density is directly obtainable

20 $$\eta \left(j\right)=U_{bias}\left(j\right)-U_{bias}^{eq}-jR_{s}$$

Thus, for metal electrodes, the Tafel plot is readily accessible. For semiconducting electrodes, however, the situation is more involved due to the fact that there is now an additional potential drop in the semiconducting material Δϕ_sc_, such that eq 18 becomes

21 $$U_{bias}-U_{ref}=\Delta \phi_{sc}+\Delta \phi_{H}+jR_{s}$$

For visual aid in the understanding, the potential drops for metal and semiconductor electrodes are schematically compared in Figure 3.

**Figure 3:**
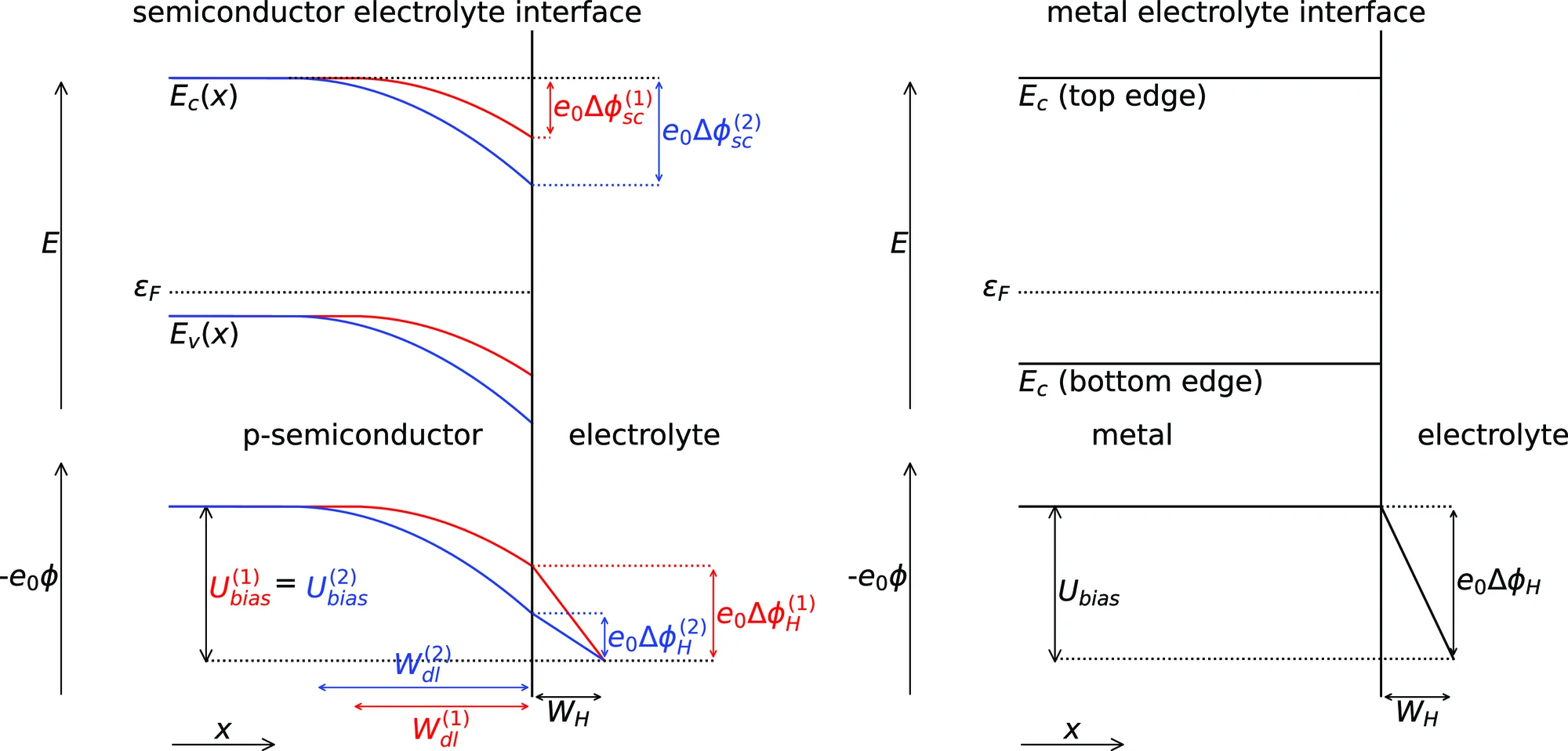
Comparison of the interface between a p-semiconducting electrode and the electrolyte (left) and the interface between a metal electrode and the electrolyte (right). For each case, the top figure sketches the relevant band edges. For the semiconductor–electrolyte interface, these are the top valence band edge *E*_*v*_(*x*) and the bottom conduction band edge *E*_*c*_(*x*). For the metal-electrolyte interface, these are the top and bottom edge of the conduction band. Also shown are the Fermi-levels, ε_*F*_, of the solid phases. In the semiconductor case, ε_*F*_ corresponds to the quasi-Fermi level of the majority carriers (holes). The bottom figure shows in both cases the dependence of the electrostatic potential, ϕ, on position. Since the interior of a metal is free of macroscopic electrostatic fields, the total potential drop *U*_bias_ for the metal-electrolyte interface occurs in the Helmholtz layer (Δϕ_*H*_), which is characterized by its width *W*_*H*_. Note that we neglect the series resistance, *R*_*s*_, of the electrochemical cell in this drawing. To be precise, we should replace *U*_bias_ by *U*_bias_–*jR*_*s*_, where *j* is the current flowing through the cell. Since the ohmic drop, *jR*_*s*_, can be determined with the help of for example EIS, we can, for simplicity and without loss of generality, consider *U*_bias_ in the sketch to be corrected by the ohmic drop. The situation changes when considering the semiconducting electrode. There, the applied bias drops in the depletion layer (Δϕ_sc_) with width *W*_dl_ in the semiconducting material and in the Helmholtz layer (note that the sketch is not to scale). Thus, *U*_bias_ = Δϕ_sc_ + Δϕ_*H*_ follows. There is no way to a priori know how large each of the two individual terms will be. Furthermore, the individual contributions may vary depending on the presence of surface states, the illumination intensity and the pH of the electrolyte.^8,9,22,44^ It is therefore possible that, unlike for metal electrodes, the same applied bias potential may correspond to different potential distributions at the semiconductor electrolyte interface. This is shown with the red and blue colours, depicting two different states (1) and (2) of the electrode with the same applied bias potential, but different distributions of the potential drop on the semiconducting material and the Helmholtz layer.

One often assumes, based on the higher capacitance of the Helmholtz layer, that the potential drop in the Helmholtz layer is constant for a given system.^28^ While this assumption may be reasonable for semiconductor electrodes operated in the dark, under illumination, band edge unpinning induced by illumination (see Figure 4) or potential-induced surface state formation/passivation is expected to also change the potential drop in the Helmholtz layer.^8,9,22,44^ Thus, in the case of an illuminated semiconducting material, the overpotential defined as in eq 19 is not obtainable simply by referencing the applied potential against the equilibrium potential (eq 20). If one compares two different current densities *j*_1_ and *j*_2_, one obtains

22 $$\eta \left(j_{2}\right)-\eta \left(j_{1}\right)=U_{bias}\left(j_{2}\right)-U_{bias}\left(j_{1}\right)-\left(j_{2}-j_{1}\right)R_{s}-\left[\Delta \phi_{sc}\left(j_{2}\right)-\Delta \phi_{sc}\left(j_{1}\right)\right]$$

**Figure 4:**
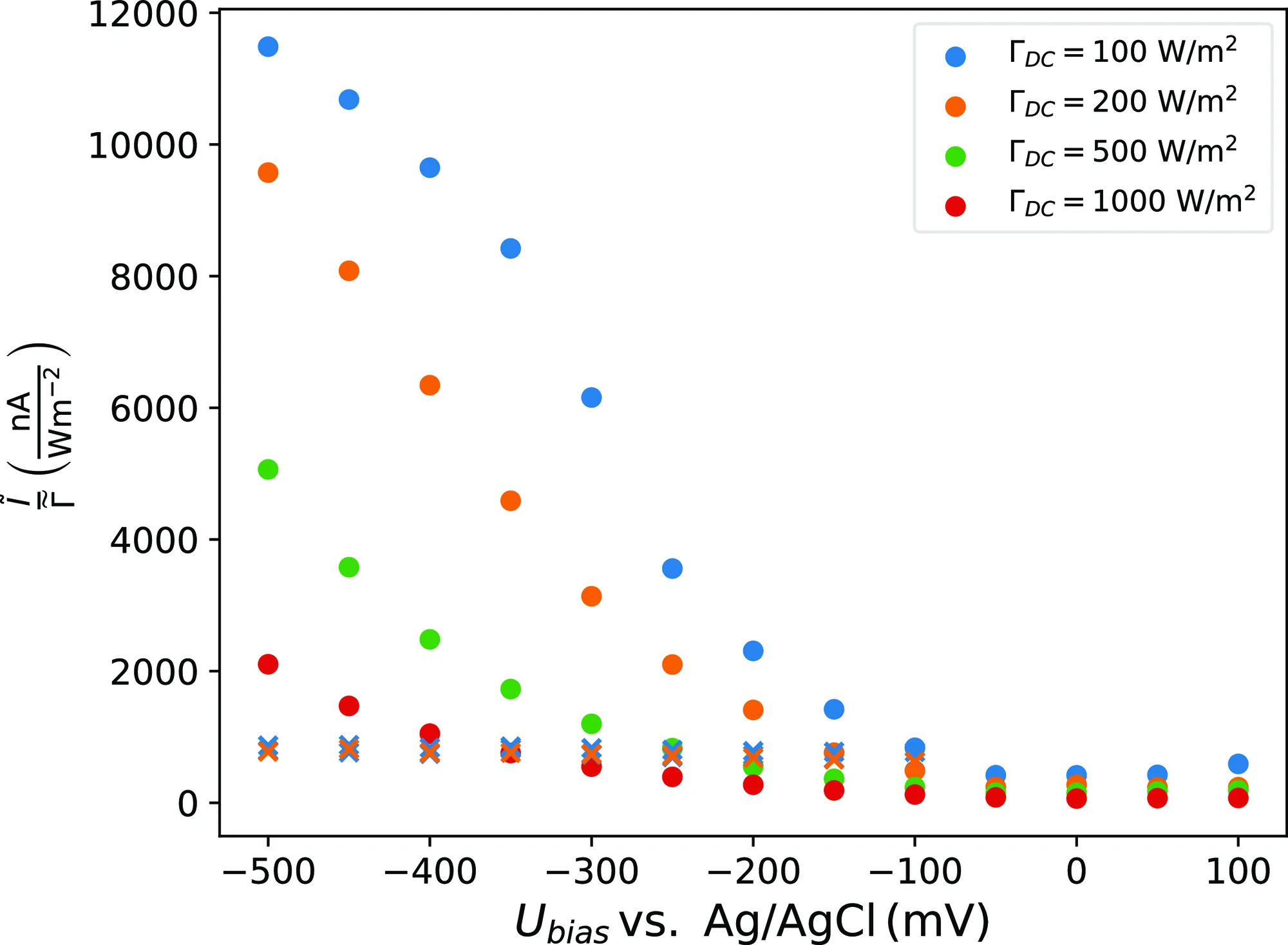
Exemplary data obtained with IMPS for a *p*-InP photoelectrode decorated with rhodium nanoparticles depicting the amplitude of the modulated minority carrier flux, Ĩ, normalised to the modulation amplitude of the illumination intensity, Γ̃, plotted against the applied bias potential, *U*_bias_. Different colours indicate different applied steady-state illumination intensities, Γ_DC_. For comparison, data points obtained for a bare *p*-InP sample are also shown as crosses. A decreasing normalised minority carrier flux with increasing Γ_DC_ for a given applied bias potential *U*_bias_ directly shows light-induced band edge unpinning. This can be understood by considering the AC Gärtner flux (eq 10) and the dependence of the depletion layer width, *W*_dl_, on band bending and thus the potential drop in the semiconducting material, Δϕ_sc_. A decreasing AC minority carrier flux corresponds to a decreasing potential drop in the semiconducting material, which implies for a stationary applied bias potential a larger potential drop in the Helmholtz layer, which in turn corresponds to the unpinning of the band edges. Since the AC minority carrier flux for the bare *p*-InP sample increases very little with applied bias, one can conclude that for the bare *p*-InP sample, a large part of the applied bias drops in the Helmholtz layer, increasing the electrochemical reaction rate to compensate for the low catalytic activity of the bare *p*-InP substrate for the HER.

Thus, to obtain the increase in overpotential corresponding to a given increase in current density, one needs to find a way to determine Δϕ_sc_(*j*_2_)–Δϕ_sc_(*j*_1_). For this task, we propose to use IMPS. As discussed above, IMPS allows one to obtain the AC minority carrier flux (scaled by a prefactor) which should show a one-to-one correspondence with band bending and thus the potential drop in the semiconducting material. When simultaneously precisely monitoring the AC illumination intensity amplitude, the IMPS data can be normalised by the AC illumination intensity. By that, the IMPS response becomes independent of the AC illumination intensity for a given light source, as long as one operates in the linear regime. In the case of the AC Gärtner flux (eq 10), this is directly obvious when dividing by the AC component of the impinging photon flux Φ̃. In Figure 4, exemplary data for the hereby determined normalised minority carrier flux for a *p*-InP photoelectrode decorated with rhodium nanoparticles is plotted against the applied bias potential. From assumptions made above, it follows that for two different applied conditions (bias potential and DC illumination intensity), for which the normalised AC minority carrier fluxes are equal, the DC band bending for these two operating points should be equal and for these given data points eq 22 reduces to

23 $$\eta \left(j_{2}\right)-\eta \left(j_{1}\right)=U_{bias}\left(j_{2}\right)-U_{bias}\left(j_{1}\right)-\left(j_{2}-j_{1}\right)R_{s}$$

Thus, the difference in overpotential for these two applied conditions is obtainable. Therefore, one finds that the change of overpotential from η­(*j*_1_) to η­(*j*_2_) corresponds to a change in the DC current density from *j*_1_ to *j*_2_. This procedure if of course directly extendable to more than two operating points corresponding to the same normalised minority carrier flux. The idea of the method is illustrated in Figure 5.

**Figure 5:**
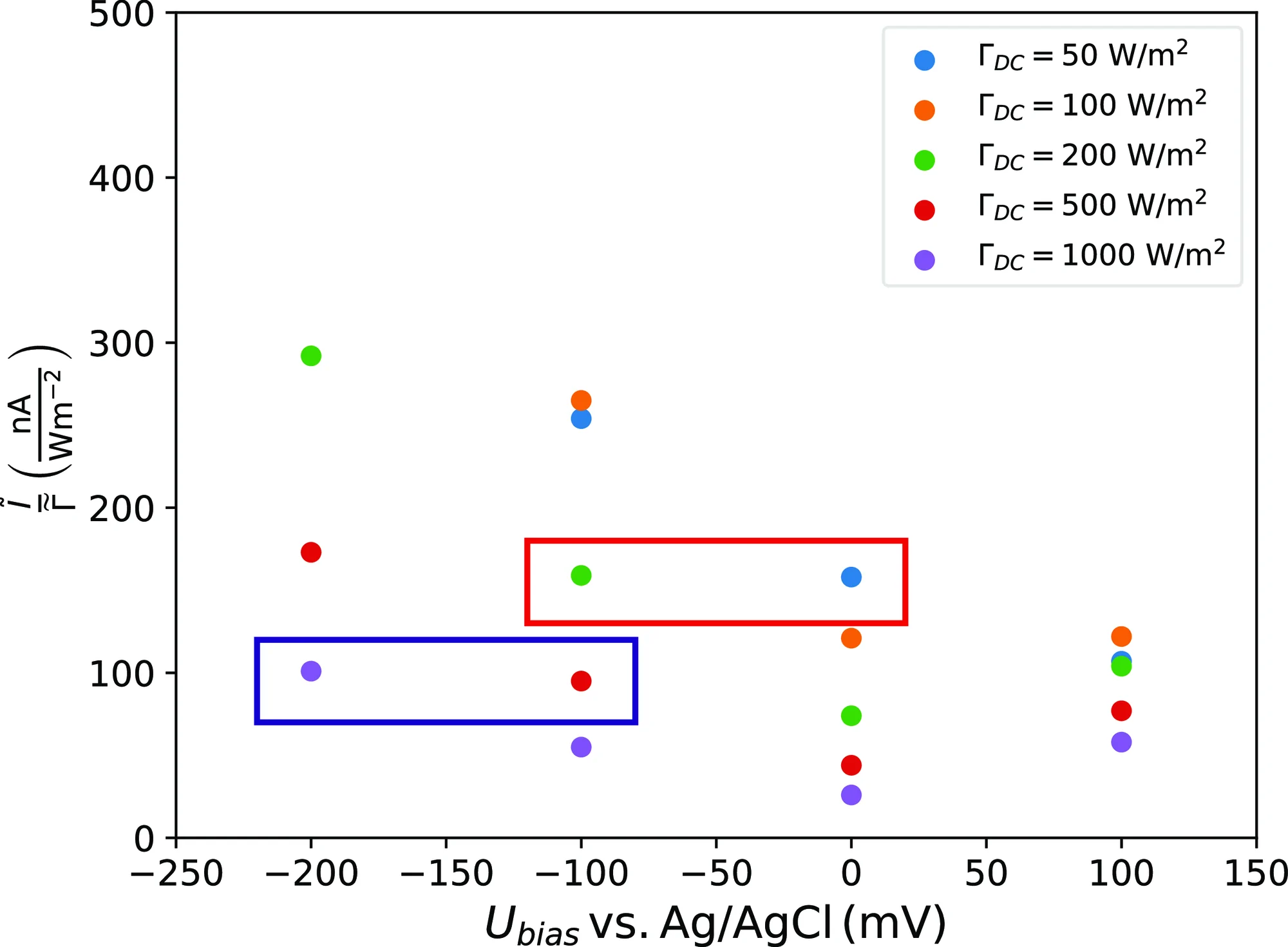
Similar plot as in Figure 4. This plot shows how different data points are correlated to each other with respect to eq 23. Highlighted with the red rectangle are two data points of different applied conditions (*U*_bias_ and Γ_DC_) that have the same normalized minority carrier flux. Thus, at these two applied conditions, the potential drop in the semiconductor, Δϕ_sc_, should be the same. The application of eq 23 then gives the difference in overpotential for these two applied conditions. The same holds for the two data points in the blue rectangle.

Considering now the construction of current density vs. overpotential plots from the hereby obtained data, an issue arises: While one knows for a given pair of data with the same normalised minority carrier flux how they are separated on the η-axis, one does not know where different pairs with different normalised minority carrier fluxes have to be placed on the η-axis in relation to each other. To solve this issue, an ordering prescription has to be proposed. The simplest and most reasonable of such ordering proposals is the assumption that a more cathodic current density corresponds to a more cathodic overpotential. Ordering the data in this manner then allows one to construct a current density vs. overpotential plot. Note though, that, since the data point corresponding to the lowest current density (in absolute values) is not obtained at η = 0, the current is plotted against a reference overpotential, η_0_, which is precisely the overpotential at the data point of lowest cathodic current density (in absolute values). How to deal with this unknown η_0_ will be discussed later. In Figure 6, a hereby constructed current density overpotential plot from the data in Figure 4 is shown.

**Figure 6:**
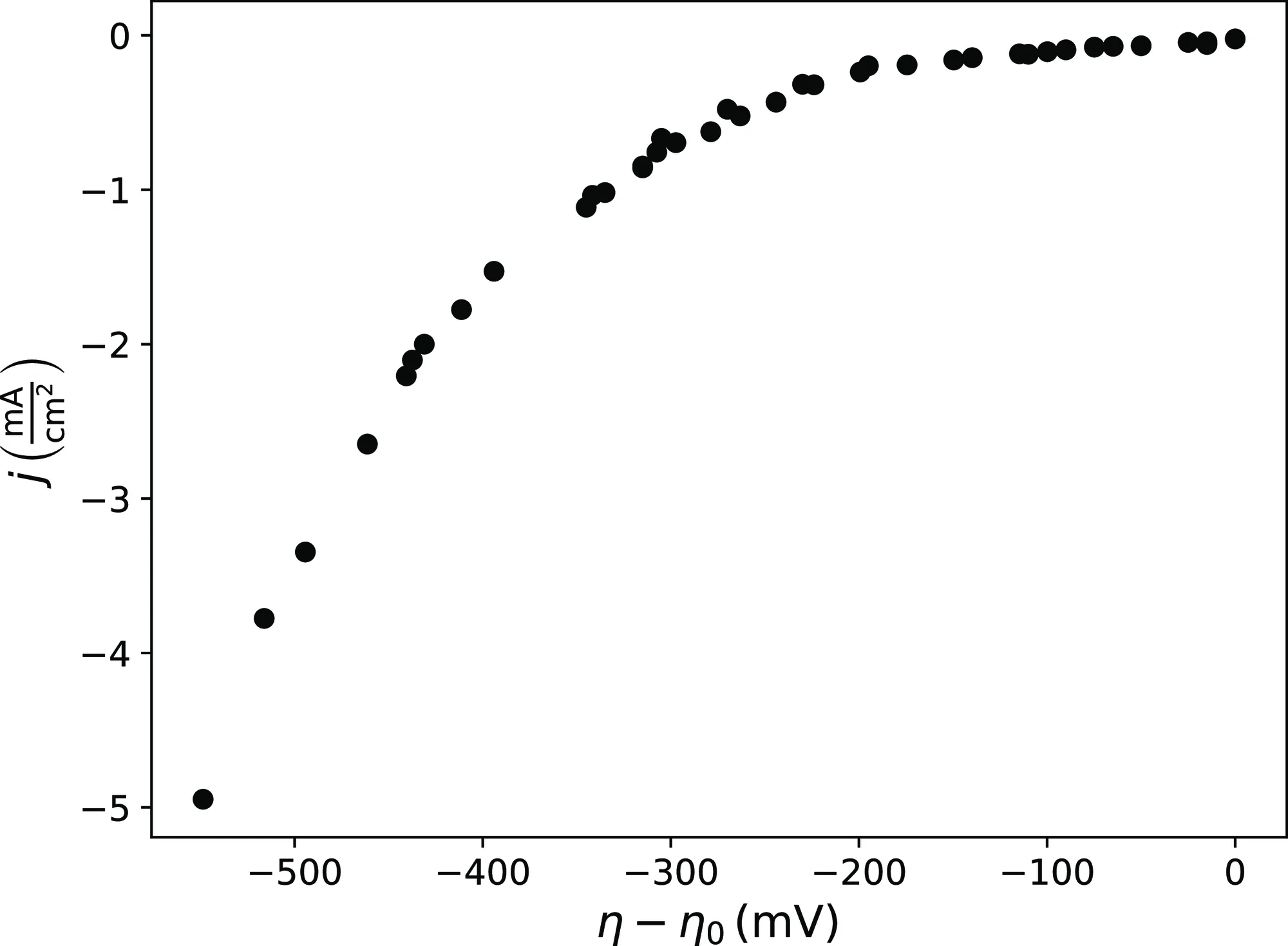
Current-overpotential plot constructed by ordering the correlated data points obtained, as described in the text, from application of eq 23 to the data shown in Figure 4. The overpotential, η, is measured against an unknown reference overpotential, η_0_.

From the constructed current density vs. overpotential plot, one may immediately construct the Tafel plot, as shown in Figure 7. Using the y-intercept of the linear regression curve (value at η–η_0_ = 0), one may evaluate an upper bound for the exchange current density. From eq 16 one obtains

24 $$j=-j_{0}\;\exp\left(-\alpha f\eta_{0}\right)\exp\left[-\alpha f\left(\eta -\eta_{0}\right)\right]$$

**Figure 7:**
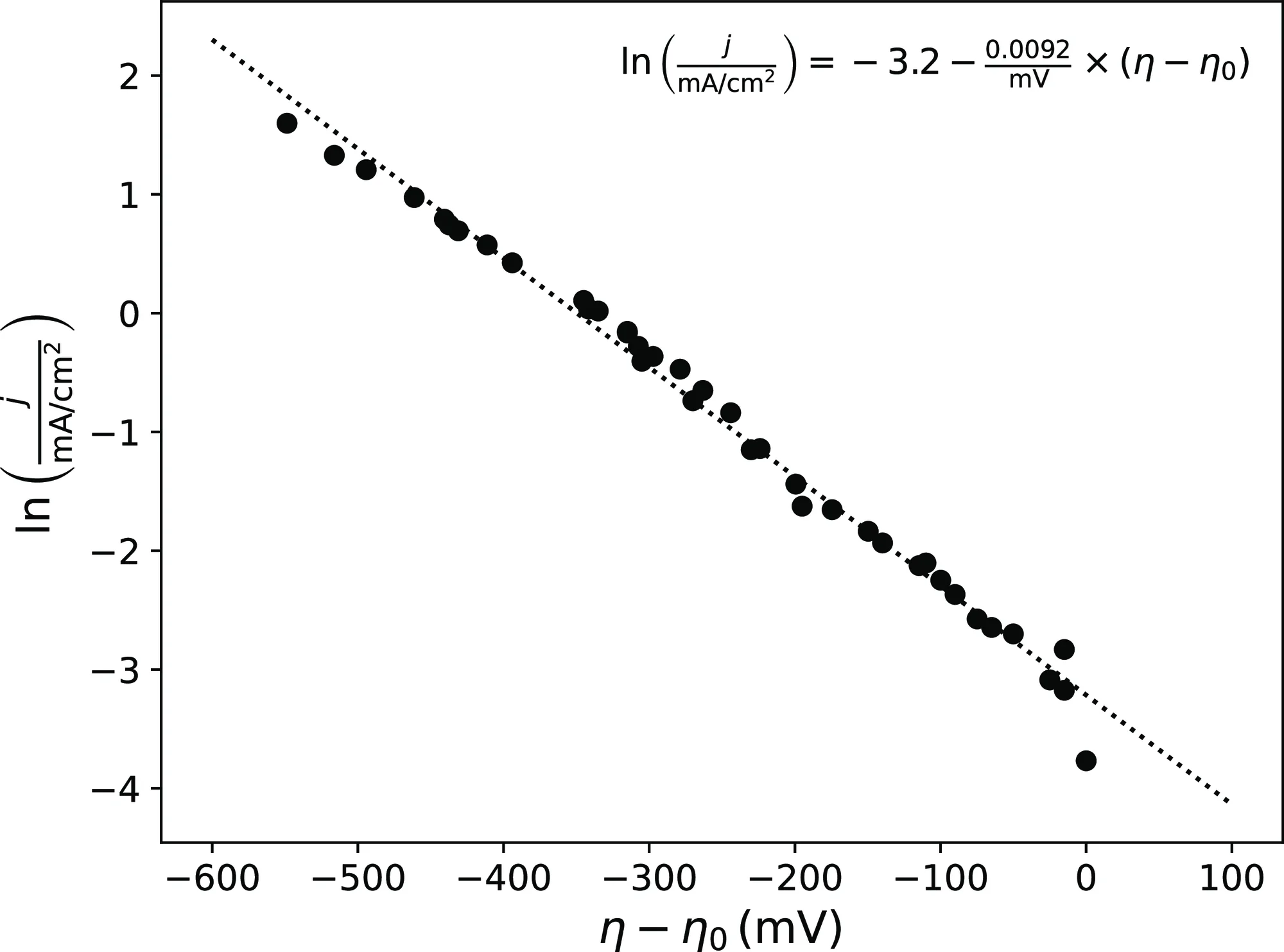
Constructed Tafel plot by taking the natural logarithm of the absolute value of the current density shown in Figure 6. The dotted line shows the linear regression curve fitted to the data. On the top right, the equation for this curve is given. The *y*-intercept directly gives the natural logarithm of *j*_0_^upper^ (in $\frac{mA}{cm^{2}}$ ) and the slope gives α*f*, as described in the text.

From that one immediately notes that, using this method, it is possible to obtain

25 $$j_{0}^{upper}=j_{0}\;\exp\left(-\alpha f\eta_{0}\right)$$

as exchange current density. Since the cathodic branch is assumed, η_0_ < 0 and therefore *j*_0_^upper^ > *j*_0_ holds. Thus, an upper bound for the exchange current density may be obtained. It is clear from eq 25 that an estimate for η_0_ is needed to further narrow down the value of the exchange current density. A crude approach is to consider the difference in applied bias potential for the lowest current density (in absolute values) and the open circuit potential (OCP) under illumination. The OCP corresponds roughly to equilibrium conditions and may thus be taken as an approximation for η = 0. This is justified if the OCP is predominantly determined by the HER, since then a zero current (OCP condition) should correspond to an equilibrium of the HER. The OCP may be expected to be determined predominantly by the HER if a facile HER is possible. For the case we are considering in this work, namely catalytically active co-catalysts (Pt/Rh) for the HER and acidic electrochemical conditions (1 M HCl), the assumption of a facile HER should hold. Using eq 22

26 $$\eta_{0}\approx U_{bias}\left[j\left(\eta_{0}\right)\right]-U_{bias}^{OCP}-j\left(\eta_{0}\right)R_{s}-\left\{\Delta \phi_{sc}\left[j\left(\eta_{0}\right)\right]-\Delta \phi_{sc}^{eq}\right\}$$

follows. From that, an upper bound (in absolute values) for η_0_ can be obtained if one neglects the series resistance and the change in the potential drop in the semiconductor from OCP to the lowest measured current density (in absolute values)

27 $$\eta_{0}^{upper}=U_{bias}\left(j\left(\eta_{0}\right)\right)-U_{bias}^{OCP}$$

Using this upper bound, one can obtain a lower bound for the exchange current density by rearranging eq 25 (note that η_0_^upper^ < 0)

28 $$j_{0}^{lower}=j_{0}^{upper}\;\exp\left(\alpha f\eta_{0}^{upper}\right)$$

Thus, one obtains a lower and an upper bound for the exchange current density

29 $$j_{0}^{lower}\leq j_{0}\leq j_{0}^{upper}$$

It is clear that one should try to record IMPS spectra as close as possible to the OCP to push *j*_0_^lower^ to the true value. This will in general be impeded by a low signal-to-noise ratio and large recombination rates close to OCP. The whole method is of course directly transferable to a photoanode making appropriate sign changes.

To obtain exchange current densities that reflect only the catalytic activity (not being influenced by the amount of loading) of a nanoparticular co-catalyst, one can normalise the obtained exchange current densities by the projected geometric area covered by the co-catalyst nanoparticles. To accomplish this, the coverage by nanoparticles can be determined using SEM imaging. A fractal structuring of the nanoparticular catalysts may in principle lead to a residual area effect on the normalised exchange current densities, as it is also the case for structured bare metal catalysts (e.g. due to surface roughening).

A short discussion about possible error sources and error propagation for the proposed method shall follow. First of all, measurement uncertainty in the IMPS response may lead to uncertainty in the determination of the normalised minority carrier flux. This may lead to erroneous paired data points after the method shown in Figure 5. Erroneous paired data points lead to an error in the assignment of the overpotential for a given current density and influence thus the quality of the linear regression used to obtain the exchange current densities. The extent of the uncertainty in the overpotential Δη due to uncertainty in normalised minority carrier flux $\Delta \frac{Ĩ}{\mathrm{\Gamma ̃}}$ depends to first order on the change of normalised minority carrier flux with band bending

30 $$\left|\Delta \eta \right|=\left|{\left(\frac{\partial \frac{Ĩ}{\mathrm{\Gamma ̃}}}{\partial \Delta \phi_{sc}}\right)}^{-1}\Delta \frac{Ĩ}{\mathrm{\Gamma ̃}}\right|$$

A similar effect arises due to a possible uncertainty in the determination of the series resistance *R*_*s*_ via EIS. An erroneous determined series resistance leads again to an error in the assignment of the overpotential for a given current density and influences thus also the quality of the linear regression. Here, the extent of the uncertainty in the overpotential depends to first order on the DC current density *j* and the uncertainty in the series resistance Δ*R*_*s*_

31 $$\left|\Delta \eta \right|=\left|j\Delta R_{s}\right|$$

Thus, if there is a significant error in the determination of the series resistance, the effect should become pronounced especially at higher current densities and one would expect to see deviating behaviour from the straight Tafel plot for high overpotentials. When normalising the exchange current densities by the loading *A*, an additional error in the normalised exchange current density Δ*j*_0_^M^ may arise due to erroneous loading determination Δ*A* via evaluation of SEM images

32 $$\left|\Delta j_{0}^{M}\right|=\left|\frac{j_{0}}{A^{2}}\Delta A\right|$$

### 2.3 Plotting the Minority Carrier Flux vs. Band Bending

Having obtained the parameters describing the catalytic activity of the co-catalyst (exchange current density and cathodic transfer constant), one can now calculate the potential drop in the semiconductor for a given DC state. If one takes the DC state with the lowest DC current density (in absolute values) as reference state (η_0_, *j* ^0^) one can calculate, for a given current density, *j*, the potential drop in the Helmholtz layer by rearranging eq 24 and using eq 25

33 $$\eta -\eta_{0}=\frac{\ln\left(j_{0}^{upper}\right)-\ln\left(|j|\right)}{\alpha f}$$

Furthermore, rearranging eq 22 one obtains

34 $$\Delta \phi_{sc}\left(j\right)-\Delta \phi_{sc}\left(j^{0}\right)=U_{bias}\left(j\right)-U_{bias}\left(j^{0}\right)-\left(j-j^{0}\right)R_{s}-\left(\eta -\eta_{0}\right)$$

where all quantities on the right hand side are now known. Thus, one can now calculate the band bending in the semiconductor for a given DC current density measured against a reference band bending. This reference band bending is precisely the band bending present at the DC condition for which the lowest DC current has flown (closest to OCP). Since the calculation of band bending can also be done for data points that were not used for the determination of the catalytic parameters of the co-catalyst (since they had no partner data points with similar AC minority carrier flux), this method provides also as a tool to verify the assumption made above that the AC minority carrier flux and band bending show a one-to-one correspondence. With the hereby obtained values for band bending, the AC minority carrier flux can be plotted against the calculated band bending and the proposed one-to-one correspondence can be checked immediately.

Therefore, equipped with the proposed evaluation scheme, one is now able to separate the total potential drop into the potential drop in the Helmholtz layer, directly influencing the activity of the co-catalyst, and the band bending in the semiconducting material, directly influencing the ability of the photoabsorber to separate light-generated electron–hole pairs.

## 3 Results and Discussion

### 3.1 Tafel Plots

The above-developed method was applied to *p*-InP photoelectrodes immersed in 1 M HCl. Beforehand, samples were functionalized by photoelectrochemically depositing rhodium or platinum nanoparticles on the surface. The functionalisation method was adapted from a procedure reported in the literature.^11^ Equipped with the method described above, one may now quantitatively estimate the catalytic properties of surfaces covered with metal nanoparticles and compare them to the bare metal electrodes. This was done for samples covered with different amounts of rhodium or platinum nanoparticles. Representative IMPS plots in the Nyquist format obtained for one of the samples are shown in Figure S1 in the Supporting Information. The fractional coverage of the surfaces with co-catalyst nanoparticles was derived from a series of SEM images. Exemplary SEM images are shown in Figures S2,S3 in the Supporting Information. The estimated exchange current densities are given in Table 1.

**Table 1:** Estimated Intervals for the Exchange Current Densities of the with Rhodium or Platinum Nanoparticles Functionalized *p*-InP Samples, Measured in 1 M HCl^a^

co-catalyst	covered area	exchange current density *j*_0_	exchange current density *j*_0_^M^
Rh	1%	$10^{-3.7}\frac{mA}{cm^{2}}\leq j_{0}\leq 10^{-2.1}\frac{mA}{cm^{2}}$	$10^{-1.7}\frac{mA}{cm^{2}}\leq j_{0}^{Rh}\leq 10^{-0.10}\frac{mA}{cm^{2}}$
	12%	$10^{-2.6}\frac{mA}{cm^{2}}\leq j_{0}\leq 10^{-1.8}\frac{mA}{cm^{2}}$	$10^{-1.7}\frac{mA}{cm^{2}}\leq j_{0}^{Rh}\leq 10^{-0.90}\frac{mA}{cm^{2}}$
	15%	$10^{-2.7}\frac{mA}{cm^{2}}\leq j_{0}\leq 10^{-1.4}\frac{mA}{cm^{2}}$	$10^{-1.9}\frac{mA}{cm^{2}}\leq j_{0}^{Rh}\leq 10^{-0.58}\frac{mA}{cm^{2}}$
	32%	$10^{-1.9}\frac{mA}{cm^{2}}\leq j_{0}\leq 10^{-0.96}\frac{mA}{cm^{2}}$	$10^{-1.4}\frac{mA}{cm^{2}}\leq j_{0}^{Rh}\leq 10^{-0.47}\frac{mA}{cm^{2}}$
Pt	7%	$10^{-1.64}\frac{mA}{cm^{2}}\leq j_{0}\leq 10^{-1.29}\frac{mA}{cm^{2}}$	$10^{-0.48}\frac{mA}{cm^{2}}\leq j_{0}^{Pt}\leq 10^{-0.12}\frac{mA}{cm^{2}}$
	13%	$10^{-1.83}\frac{mA}{cm^{2}}\leq j_{0}\leq 10^{-1.17}\frac{mA}{cm^{2}}$	$10^{-0.94}\frac{mA}{cm^{2}}\leq j_{0}^{Pt}\leq 10^{-0.28}\frac{mA}{cm^{2}}$
	46%	$10^{-1.25}\frac{mA}{cm^{2}}\leq j_{0}\leq 10^{-0.27}\frac{mA}{cm^{2}}$	$10^{-0.92}\frac{mA}{cm^{2}}\leq j_{0}^{Pt}\leq 10^{0.06}\frac{mA}{cm^{2}}$

a The first column gives the type of co-catalyst nanoparticles deposited on the photoelectrode’s surface. The second column gives the percentage of the area of the surface of the p-InP substrate covered by co-catalyst nanoparticles. The coverage was determined using SEM imaging. The third column gives the exchange current densities *j*_0_ estimated after eq 29. The fourth column gives the exchange current densities referenced to the area covered by co-catalyst nanoparticles *j*_0_^M^.

As expected, the exchange current density increases with rhodium or platinum coverage, as seen in the third column of Table 1. In the fourth column of Table 1, the exchange current densities are referenced to the area covered by rhodium or platinum nanoparticles. Note that here, the estimated intervals for the hereby normalised exchange current densities are now very similar for all the samples with the same co-catalyst material. This indicates that the effective exchange current density is linearly dependent on the area covered by rhodium or platinum nanoparticles (at least for the amounts deposited here). These values should allow for a comparison of the catalytic activity of the deposited nanoparticles with bare rhodium or platinum metal electrodes. For bare rhodium metal electrodes, exchange current densities between $10^{-0.6}\frac{mA}{cm^{2}}$ and $10^{-0.2}\frac{mA}{cm^{2}}$ and for bare platinum metal electrodes, exchange current densities between $10^{-0.53}\frac{mA}{cm^{2}}$ and $10^{0.00}\frac{mA}{cm^{2}}$ have been reported in the literature.^45,46^ Comparing with the upper boundaries shown in the fourth column of Table 1, the deposited nanoparticles and the corresponding bare metals seem to show a comparable catalytic activity for the HER, though the rhodium nanoparticles seem to tend to have a little bit more sluggish kinetic behaviour than the bare rhodium metal. In the next section it is shown that the lower bounds for the exchange current densities are probably quite far away from the exact value, indicating that the exact value may lie nearer to the upper bound. Comparing the obtained values for rhodium and platinum, the trend from the bulk materials that platinum shows a bit higher exchange current density is reproduced for the nanoparticles.

Considering the cathodic charge transfer coefficients, α, we obtained similar values of α = 0.21–0.41 for rhodium and platinum as co-catalyst. Interestingly, these values differ greatly from the reported values in literature for bare rhodium and platinum metals of about α = 2.^45,47^ It was lately discussed by Prats and Chan^48^ that the reported values of α = 2 may very likely be disturbed by mass transport limitations due to the facile HER at these metals. They proposed that α ≈ 0.5, which fits quite well to our results, seems to be a more reasonable value if mass transport limitations are excluded. Since the nanoparticular and disentangled nature of the metal particles in our case may reduce mass transfer limitations drastically,^49^ the obtained small values for α may be explainable by this effect.

Note that no saturation of the solution with hydrogen gas was conducted beforehand. Since the exchange current density is dependent on the concentrations of the corresponding redox pair,^41^ this may explain the seemingly more sluggish kinetic behaviour of the rhodium nanoparticles compared to the values for rhodium metal electrodes given in the literature. In fact, smaller concentrations of the reduced species (hydrogen gas) will shift the equilibrium potential anodically. This corresponds directly to a smaller exchange current density. The error should not be too large, though, since very large changes in activity of the redox species are required to produce significant changes.

Furthermore, a different morphology of the catalyst layer may lead to different exchange current densities, since the true area will be increased in the case of micro-/nano-structures, and possibly even the nature of the catalytically active sites. In the case of micro-/nano-structures, a higher exchange current density is obtained, when referencing to the projected geometric area covered by the catalyst layer, yet this will also change to some extent with the loading, as particle growth modes are expected to change. Note that the interval for the hereby estimated exchange current densities is about up to 1 order of magnitude wide. Since reported exchange current densities for the HER at metal electrodes span roughly 10 orders of magnitude,^45,46^ this estimate should still allow for semi-quantitative discussions regarding the efficiency of functionalized photoelectrode surfaces.

### 3.2 Minority Carrier Flux vs. Band Bending

Having obtained the catalytic parameters of the co-catalyst, one can now calculate the band bending at a given operational point after eq 34 and correlate it with the normalised AC minority carrier flux. This is exemplary shown in Figure 8 for one of the *p*-InP samples covered with platinum as co-catalyst. As can be clearly seen, there is a one-to-one correspondence between AC minority carrier flux and calculated band bending. Since we also used complementary data points that were not used to obtain the catalytic parameters for this construction, the obtained plots are direct experimental support for the assumption of the whole method. Furthermore, one also clearly sees, that there is no dependence on the DC illumination intensity. Only for small band bending, there seems to be an outlier for $\Gamma_{DC}=50\frac{W}{m^{2}}$ . A possible reason for the occurrence of the outlier may be the smaller signal-to-noise ratio for small values of the band bending and small AC illumination intensity (10% of the DC illumination intensity) due to a smaller photocurrent and thus smaller signal.

**Figure 8:**
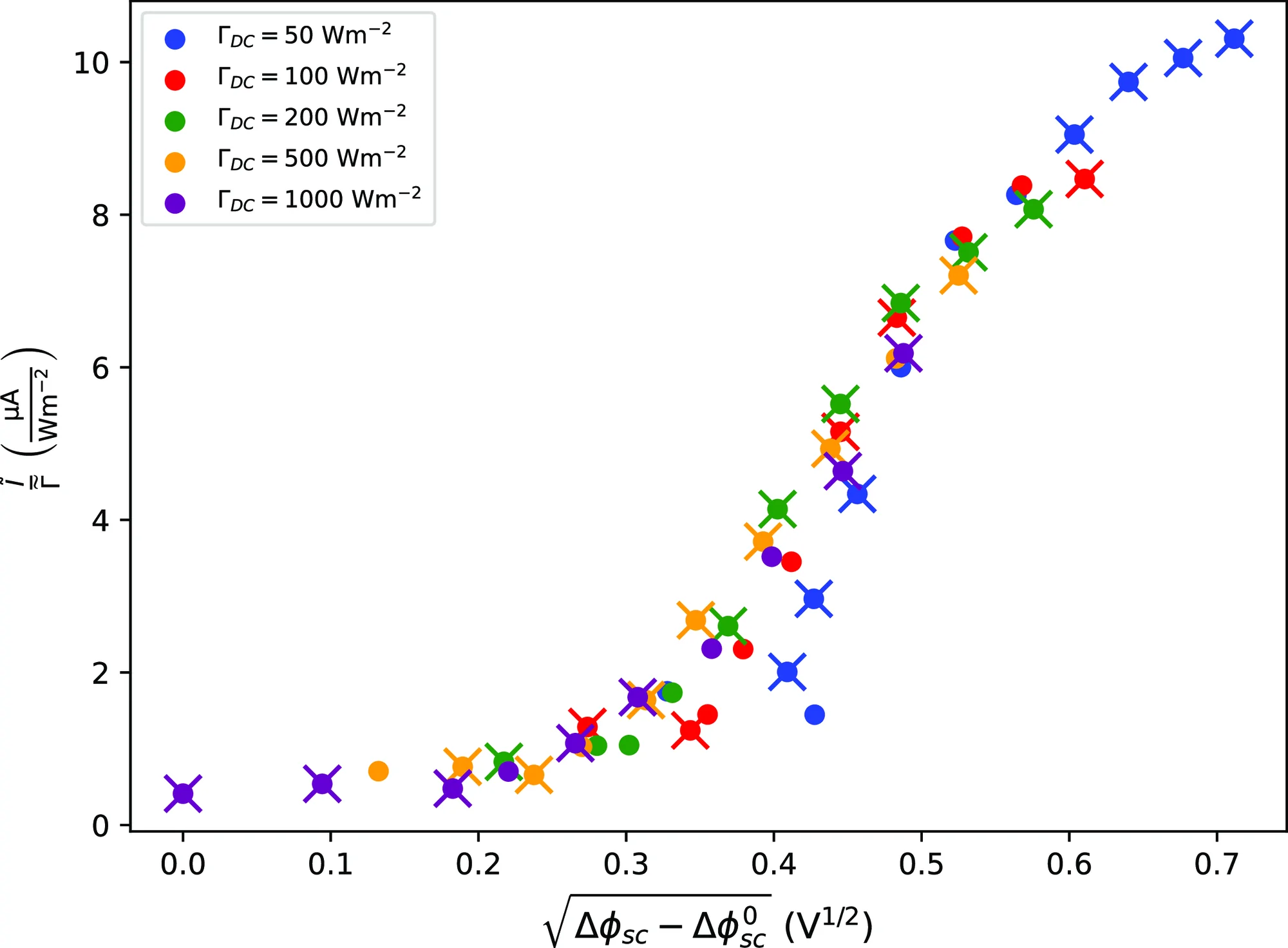
Normalised AC minority carrier flux as determined by IMPS for a *p*-InP photoelectrode covered with platinum nanoparticles as co-catalyst, plotted against the square root of the calculated band bending. The different colours indicate the steady-state illumination intensity Γ_DC_ applied during the IMPS measurement, corresponding to the given data point. The band bending was calculated after eq 34 using the beforehand determined parameters α and *j*_0_^upper^. Data points not used to obtain the catalytic parameters are highlighted by a cross.

Note that in Figure 8, the AC minority carrier carrier flux was plotted against the square root of the calculated band bending to compare the obtained plot with the Gärtner flux (eq 10). The Gärtner flux is dependent on the width of the depletion layer, which in turn should be proportional to the square root of band bending (eq 12).^27,28^ Our obtained plots do, however, not qualitatively match with the expected plot of a Gärtner-type flux,^29^ especially in the low band bending region. The Gärtner flux does not show the here obtained change of curvature in the low band bending region. Note that a deviation of the minority carrier flux from the Gärtner flux may be expected, especially in the low band bending regime. There, electron–hole pair recombination in the depletion layer may not be expected to be negligible.^50^ Neglecting the electron–hole pair recombination in the depletion layer is precisely the main assumption in the derivation of the Gärtner flux.^29^ Thus, our results indicate that the Gärtner flux does not in general give a good qualitative approximation for the AC minority carrier flux of the IMPS measurement.

Reactive semiconductor surfaces, such as InP, can very sensitively react to defects already introduced during growth or the electrochemical conditioning. The latter can be adsorbates, surface states forming or passivating in narrow potential windows, or even the periodic evolution and dissolution of surface phases under fixed conditions over time leading to emergent complexity of the interface.^51^ As a consequence, slight variations in the experimental parameter space, a given sample has experienced, might lead to significant variations in the surface state density, which in turn impacts interfacial energetic alignments and minority carrier fluxes. The approach developed above now allows us to quantitatively assess minority carrier fluxes for different samples.

In Figure 9, we compare the obtained AC minority carrier flux vs. band bending plots for different *p*-InP samples covered by varying amounts of platinum nanoparticles. There, the AC minority carrier flux is normalised to unity to exclude differences due to varying sample size and parasitic light absorption/reflection by the platinum co-catalyst nanoparticles. Note firstly that also here, the AC minority carrier flux clearly increases with increasing band bending for all the samples. Furthermore, it is of interest to discuss differences in the plots for the different samples. In principle, one would expect this plot to be the same for a given photoelectrode material, regardless of the amount of co-catalyst deposited on the surface of the photoelectrode. This is due to the fact, that the amplitude of the AC minority carrier flux should only reflect the ability of the light-absorbing semiconducting material to generate and separate electron–hole pairs. While this is the case for samples 3, 4 and 5, the other three samples differ. One obvious reason for this may be the fact that we can not calculate the absolute band bending, but only the band bending referenced against the band bending at the operational point with the lowest current density, Δϕ_sc_^0^ (compare with eq 34). This reference point will not be the same for all samples.

**Figure 9:**
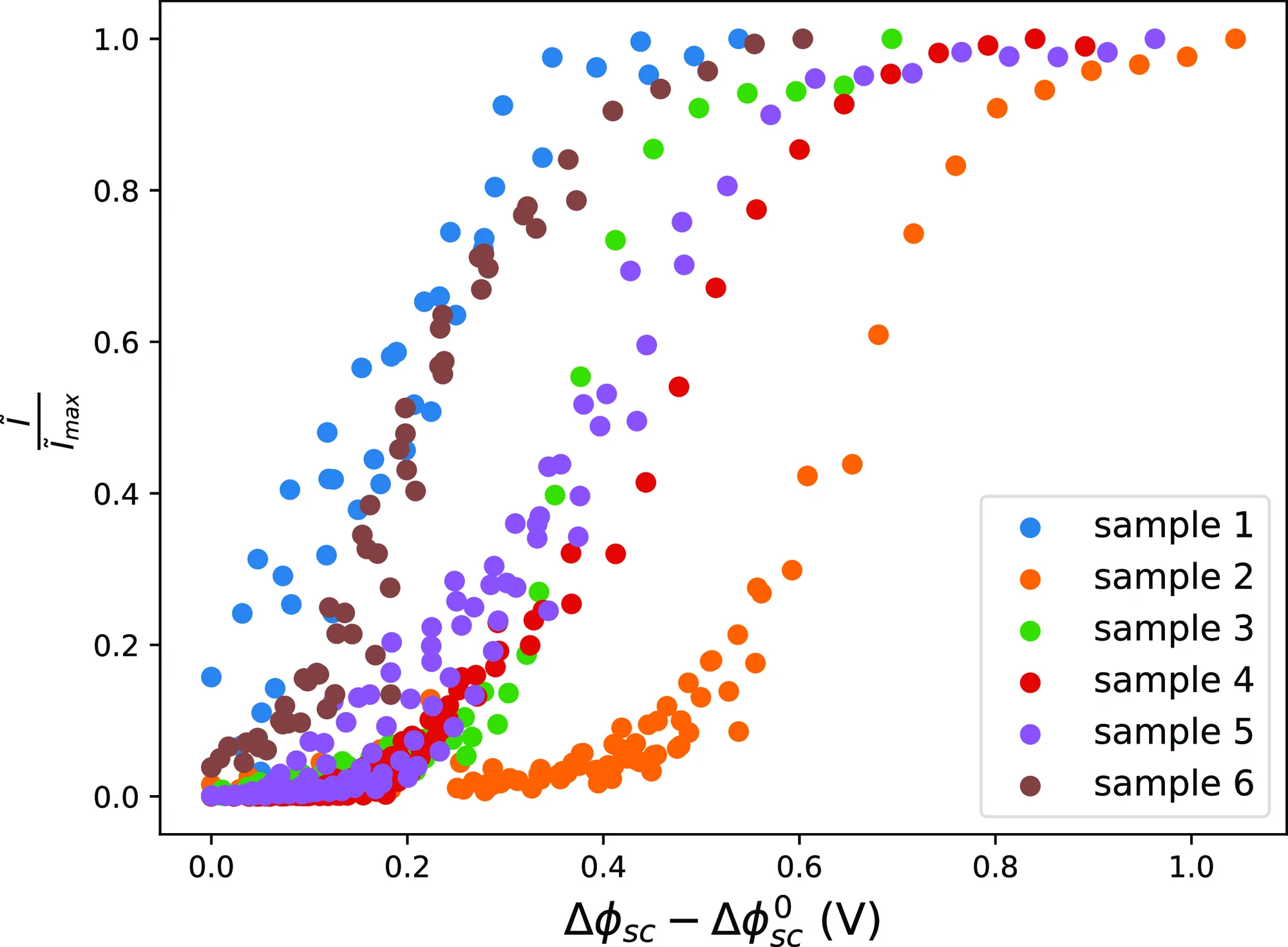
AC minority carrier flux as determined by IMPS normalised to unity for *p*-InP photoelectrodes covered with varying amount of platinum nanoparticles as co-catalyst plotted against calculated band bending. The band bending was calculated after eq 34 using the beforehand determined parameters α and *j*_0_^upper^.

Consider, for example, the OCP under $50\frac{W}{m^{2}}$ illumination intensity obtained for sample 1, compared to samples 3–5. For samples 3–5, the OCP was similar (between +0.32 V and +0.44 V vs. Ag/AgCl) while the OCP for sample 1 was much more cathodic (−0.41 V vs. Ag/AgCl). The reason for that may be formation of surface states for sample 1 in the pretreatment (CV cycling in 1 M HCl) and consequently a higher surface electron–hole recombination rate. These surface states may, for example, be present due to the formation of metallic indium at the surface of the InP sample.^11^ Then, the photovoltage produced by the photoelectrode would be smaller and the band bending at OCP (under illumination) would be larger since the photovoltage compensates the band bending for a photoelectrode working in depletion layer mode.^52^ This may explain why the curve for sample 1 seems to be left-shifted by about 0.2 V, but similar in form compared to the samples 3–5, since the reference band bending would be larger for sample 1.

For sample 2, the plot interestingly firstly follows the ones obtained for samples 3–5, but shortly above Δϕ_sc_-Δϕ_sc_^0^ = 0.2 V, the AC minority carrier flux drops and the curve seems to start off again, now right-shifted by about 0.2 V. A possible explanation for that may be a flatband shift for sample 2 due to possibly some corrosion reaction and/or formation of surface states in operando. This may then lead to the observed hysteresis behaviour.

Lastly, sample 6 has to be discussed. For this sample, the curve seems to start off in a similar fashion as the ones for samples 3–5, but the AC minority carrier flux increases more steeply with increasing band bending for sample 6. Interestingly, for this sample, there was also an increase in efficiency of the bare *p*-InP sample while preconditioning (before the platinum deposition) observable in the obtained CVs. This increase in efficiency is known from literature,^11^ but was only observed for this one single sample in our experiments. In literature it was hypothesized that a thin, hydrogen-doped n-type In_2_O_3_ (mixed with other phosphate/oxide species) layer grows while preconditioning on the surface of the photoelectrode creating a buried p-*n*-junction.^53^ The p-*n*-junction may improve electron–hole pair separation in the photoelectrode and thus lead to the observed increase in efficiency.^54^ Our data may tentatively support this hypothesis due to steepened dependence of the AC minority carrier flux on band bending.

Let us now consider the slow increase of the AC minority carrier flux with band bending for small band bending values observed for most of the samples in Figure 9. Here, the assumption of negligible change in band bending from OCP to the operational point with the lowest current density, leading to the lower bound for the exchange current density (eq 27), seems to be a very harsh approximation for our samples. Since the AC minority carrier flux reflects the ability of the semiconducting material to generate a photocurrent, our data indicate that an increase in photocurrent close to OCP has to correspond to a significant increase in band bending. Thus, η_upper_ is very likely significantly overestimated and therefore *j*_0_^lower^ significantly underestimated compared to the exact values.

## 4 Conclusions and Outlook

In summary, this work shows how to separate the total potential drop for a photoelectrode decorated with nanoparticular co-catalyst into the component in the semiconducting material–or rather its interface to co-catalyst or electrolyte–and the component in the Helmholtz layer. Through that one can derive estimates for exchange current densities for the nanoparticular co-catalyst, where Tafel plots cannot be directly derived as for bare metal catalysts. A comparison of exchange current densities from the nanoparticles on the semiconductor with the bulk metal electrode may allow to quantify morphology-dependent catalytic activities, which can vary significantly with shape for a given material.^55^ This may enable a more target-oriented design of nanoparticles as co-catalysts on photoelectrodes. Another reason for differing catalytic behaviour of nanoparticles and bulk metal may arise due to the different interfacial properties of a metal nanoparticle semiconductor junction compared to a bulk metal substrate. Strong deviations of the catalytic activity of the nanoparticles from the bulk metals may thus hint at a strong influence of the interface. Furthermore, the dependence of the ability of the semiconducting material to separate light-generated electron–hole pairs on band bending can be resolved. This enables one to analyse the effect of different preconditioning/synthesis routines on the ability of the semiconducting material to generate a large minority carrier flux which is the prerequisite for a large photocurrent and photovoltage.

Our results indicate that our method may allow for the determination of catalytic parameters even in the case of very facile reactions like the HER at platinum. Here, catalytic parameters obtained at bulk electrodes may be entangled with mass transfer limitations.^48^ Since our method allows to examine nanoparticular and disentangled catalysts, the role of mass transfer effects can be diminished considerably.^49^

Lastly, we found experimental evidence that, as in general expected,^50^ the Gärtner flux is not a good qualitative approximation for the minority carrier flux in the region of low band bending, at least for *p*-InP photocathodes. This should be kept in mind when using the Gärtner flux to fit data to obtain for example diffusion lengths^37^ or flatband potentials.^13^

## 5 Experimental Section

Electrochemical measurements were performed using a Zahner Zennium Pro potentiostat and a Zahner PP211 potentiostat was used to control the IMPS setup (Zahner). A white-light LED was used as light source for the IMPS measurements (LSW-2, provided by Zahner). Potentials are referenced against Ag/AgCl (3 M), the counter electrode was a platinum wire. Electrochemical experiments were conducted in custom-made glass cells. *p*-InP­(100) samples from InPACT (Zn-doped with doping concentration of 4 × 10^18^ cm^–3^, epi-ready, 0.1° offcut (100), thickness of 600 μm) were contacted on the backside to a wire using silver conducting paint and GaIn eutectic. Preparing the *p*-InP samples was done in reference to a procedure described in the literature.^11^ First, a pre-treatment in aqueous 1 M HCl was conducted by performing cyclic voltammetry for 1 h in the potential window [−1.5, +0.5]V vs. Ag/AgCl with a scan rate of $50\frac{mV}{s}$ and an illumination intensity corresponding to 100% AM1.5G (simulated with a Wavelabs Sinus-220 solar simulator). Afterwards, photoelectrodeposition of rhodium/platinum nanoparticles was performed in an aqueous 0.5 mM RhCl_3_/0.5 mM K_2_PtCl_6_ solution, containing additionally 0.5 M KCl/0.1 M KCl as supporting electrolyte and 2% isopropyl alcohol. The applied illumination intensity for the photoelectrodeposition was 100% AM1.5G. The applied bias potential for the rhodium deposition was *U*_bias_ = −0.2 V vs. Ag/AgCl, while for the platinum deposition, a constant current of −50 μA was applied. Deposition was done for different time spans to control the amount of deposited rhodium/platinum nanoparticles. The IMPS setup of Zahner (CIMPS) allowed to precisely control the AC amplitude of the illumination intensity and allowed thus for a normalisation by the AC illumination amplitude. IMPS measurements at different applied bias potentials and steady-state illumination intensities were done in aqueous 1 M HCl to obtain the normalised minority carrier fluxes. The modulation depth of the illumination intensity for IMPS was set to 10% of the steady-state illumination intensity. From our laboratory experience with the *p*-InP samples, the assumption of linearity of the IMPS response (small signal approximation) holds at least up to the 10% perturbation depth of the illumination used in this work. Normalised minority carrier fluxes were treated to be of equal size if their relative difference was less than a specified threshold. This threshold was fixed for a given sample and varied for the different samples between 1% and 5%. There is a trade-off between accurate determination of data points exhibiting the same normalised minority carrier flux (small threshold) and obtaining enough data points to be able to unambiguously construct the current–overpotential curve from the paired data points (large threshold). The analysis of the area covered by deposited rhodium nanoparticles was done using SEM imaging. EDX measurements confirmed the deposited particles to consist of rhodium/platinum. The series resistance of the electrochemical cell *R*_*s*_ was determined using EIS as 3–4 Ω. Neglecting the series resistance in the method used in this work did not lead to any significant effects. Therefore, the series resistance was treated as being zero for the data presented here.

## Supplementary Material



## Data Availability

The data underlying this study is openly available on Zenodo^56^ at DOI:10.5281/zenodo.22746285.

## Notes

 Figure S1: Experimental IMPS Nyquist plots of a *p*-InP sample decorated with rhodium nanoparticles; Figure S2: SEM image of rhodium nanoparticles on a *p*-InP sample (high loading); Figure S3: SEM image of rhodium nanoparticles on a *p*-InP sample (low loading) (PDF)
