# Supplementary material for: Exchange Current Densities of and Minority Carrier Fluxes to Catalyst Nanoparticles on Photoelectrodes from Intensity Modulated Photocurrent Spectroscopy
Source: ACS Meas Sci Au. 2026 Oct 1;6(5):1929–43. doi: 10.1021/acsmeasuresciau.6c00287 (PMC13693261; doi:10.1021/acsmeasuresciau.6c00287)
Supplement: Supplementary file 1 [file tg6c00287_si_001.pdf]

Supporting Information: Exchange Current Densities of and Minority Carrier Fluxes to Catalyst Nanoparticles on Photoelectrodes from Intensity Modulated Photocurrent Spectroscopy

Max Nusshör[1], Eric Hinssen[1], Maximilian Diecke[1], Erica A. Schmitt[1], and Matthias M. May*[1,2]

[1]Institute of Physical and Theoretical Chemistry, Universität Tübingen, 72076 Tübingen, Germany

[2]Center for Light-Matter Interaction, Sensors and Analytics LISA+, Universität Tübingen, 72076 Tübingen, Germany

*Email: matthias.may@uni-tuebingen.de

IMPS Nyquist plots

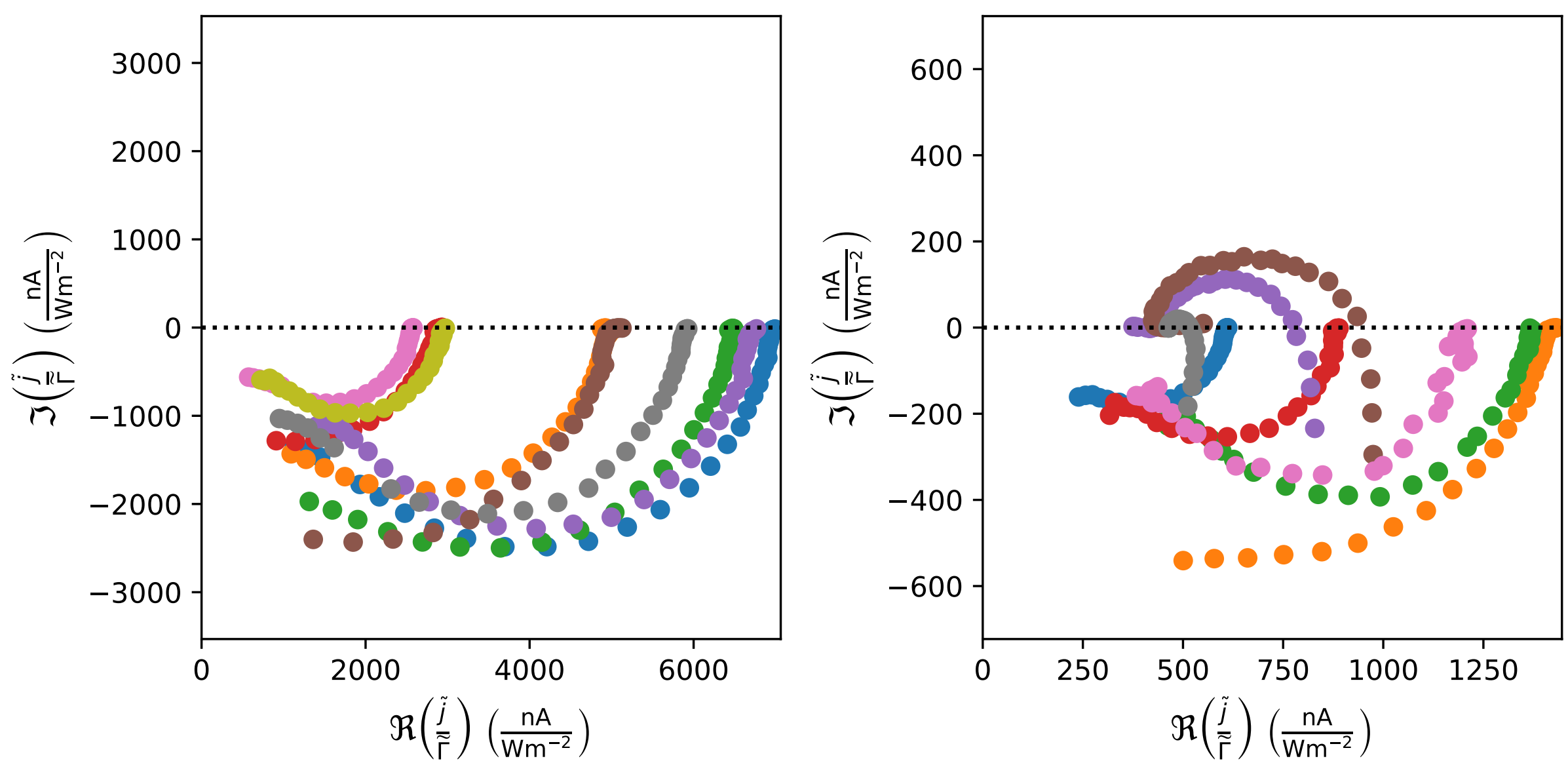

Figure S1: Exemplary Nyquist plots of a p-InP photoelectrode decorated with rhodium nanoparticles as co-catalysts used to determine the normalised minority carrier flux as described in the main text. Each colour corresponds to different applied conditions (DC illumination intensity and applied bias potential). The data is shown in two separate plots to disentangle the representation for small photocurrents. The real and imaginary parts of the photocurrent $\widetilde{j}$ are given normalised by the AC illumination intensity depth $\widetilde{\Gamma}$. Data points for frequencies of 1 Hz – 50 kHz are shown.

SEM images

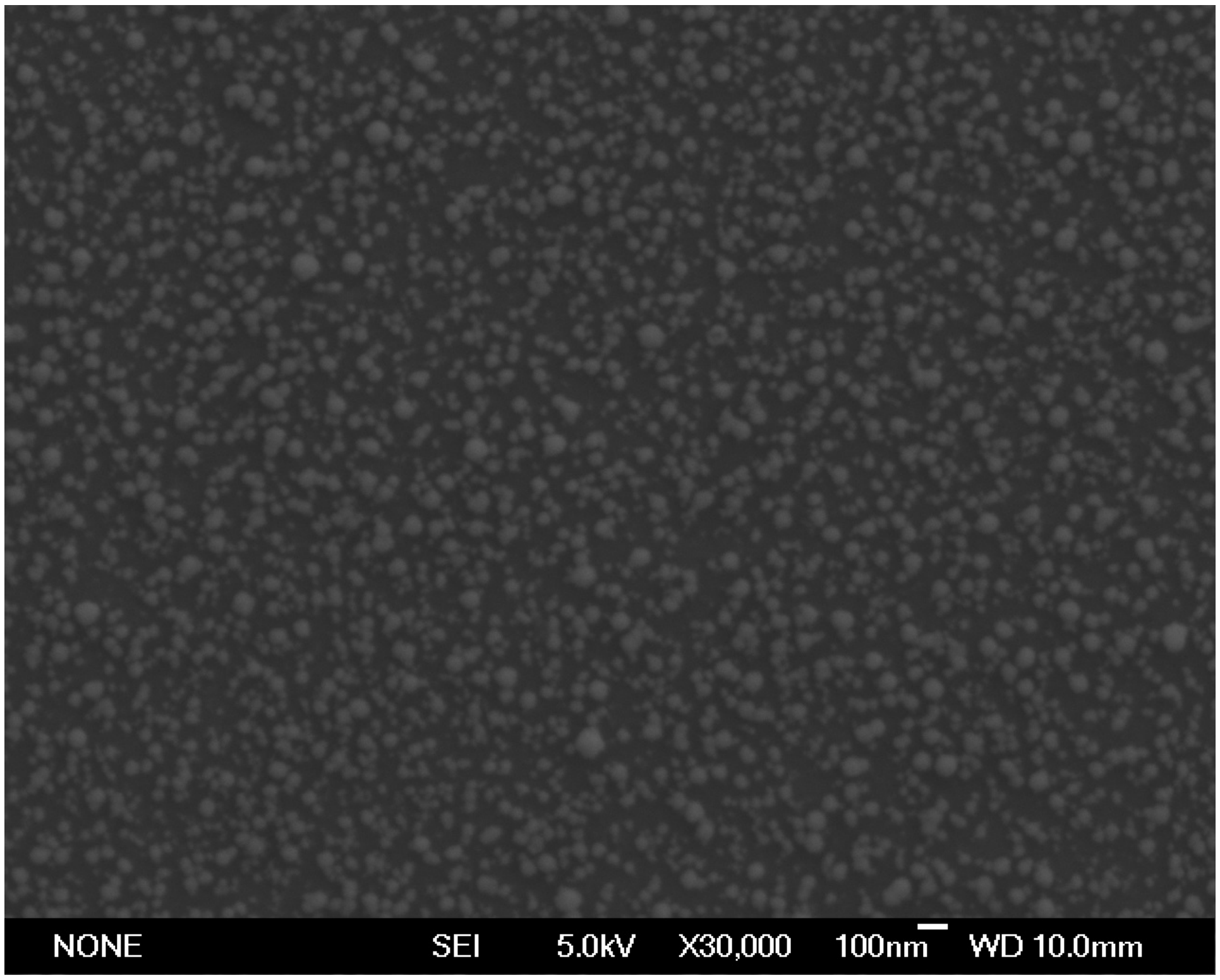

Figure S2: Exemplary SEM image of rhodium nanoparticles on the surface of a p-InP photoelectrode with a high loading.

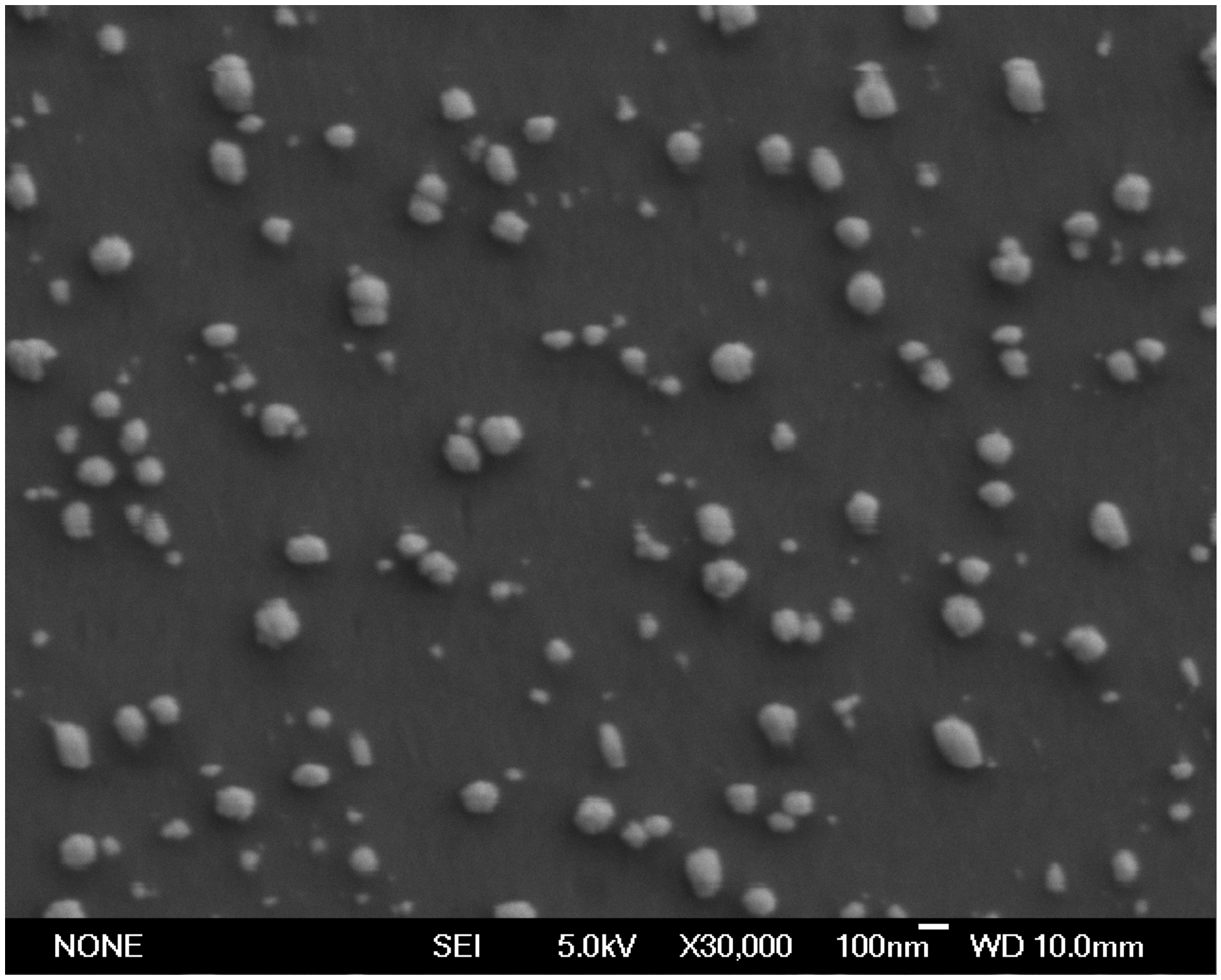

Figure S3: Exemplary SEM image of rhodium nanoparticles on the surface of a p-InP photoelectrode with a low loading.
